# Redox-driven speciation and colloid formation contribute the in vivo chemistry and organ deposition of Astatine-211

**DOI:** 10.1186/s41181-026-00441-3

**Published:** 2026-03-31

**Authors:** Xiaoxu Dong, Jiaojiao Li, Mumei Chen, Kaiqi Zhang, Xiaozheng Zhang, Fengling Shan, Shuai Xue, Xiao Li, Lan Zhang, Huawei Cai, Jiajun Liu, Qingnuan Li

**Affiliations:** 1 Shanghai Institute of Applied Physics, Chinese Academy of Sciences, Shanghai, 201800 China; 2 Department of Nuclear Medicine, Shanghai Pudong Hospital, Fudan University Pudong Medical Center, Shanghai, 201399 China; 3 Department of Nuclear Medicine and Clinical Nuclear Medicine Research Lab, West China Hospital, Sichuan University, Chengdu, 610041 Sichuan China; 4 Department of Nuclear Medicine, Ruijin Hospital, Shanghai Jiao Tong University School of Medicine, Shanghai, 200025 China

**Keywords:** Astatine-211, Radiochemical speciation, Radiopharmaceutical stability, Targeted alpha therapy, Redox-driven speciation, Colloid formation

## Abstract

**Background:**

Astatine-211 (^211^At) is a promising radionuclide for targeted alpha therapy (TAT), yet its clinical translation is hindered by poor in vivo stability and pronounced non-target organ accumulation. The fundamental radiochemical mechanisms governing the biodistribution of free ^211^At remain insufficiently understood, limiting rational radiopharmaceutical design.

**Results:**

Comparative in vivo studies revealed that free ^211^At and free iodine-131 (^131^I) initially share similar distribution patterns but diverge markedly thereafter, with ^211^At exhibiting pronounced accumulation in the stomach, lungs, liver, and spleen. Experimental speciation analyses demonstrated rapid oxidation of At^−^ to cationic intermediates in biological environments, followed by hydrolysis and colloid formation. Ultrafiltration and chromatography confirmed the generation of macromolecular species responsible for hepatosplenic retention. Co-administration of ascorbic acid (AA) partially suppressed abnormal organ uptake, supporting a redox-dependent mechanism. Density functional theory (DFT)-based Pourbaix analysis further corroborated the thermodynamic instability of At^−^ under physiological conditions and predicted the predominance of oxidized and hydrolyzed species.

**Conclusions:**

These results identify redox-driven speciation and subsequent hydrolysis/aggregation as key chemical processes contributing to the in vivo behavior of free ^211^At. Understanding and controlling astatine redox chemistry is therefore critical for improving the stability, safety, and translational development of ^211^At-based radiopharmaceuticals.

**Supplementary Information:**

The online version contains supplementary material available at 10.1186/s41181-026-00441-3.

## Background

Targeted alpha therapy (TAT) is a potent modality for precision oncology, leveraging the high linear energy transfer (LET) of α particles to induce irreparable DNA double-strand breaks (DSBs) in target cells (De Sio et al. 2025). Among the established radionuclides for TAT, free astatine-211 (^211^At, t_1/2_ = 7.2 h), administered as unbound astatide analogously to radioiodine for thyroid targeting, has emerged as a viable therapeutic candidate, potentially surpassing the efficacy of the established beta-emitter iodine-131 (^131^I, t_1/2_ = 8.0 d) (Ansari-Chauveau et al. 2025; Frost et al. 2013; Watabe et al. 2025). For instance, in murine models of differentiated thyroid cancer, a single dose of free ^211^At or ^211^At-based therapy achieved complete regression of the primary tumor and its metastases at activities as low as 40 kBq, and extended median survival beyond 120 days, effects unmatched by ^131^I at comparable doses, while inducing only limited and reversible hematotoxicity (Vaidyanathan et al. 1994; Wang et al. 2020; Watabe et al. 2025; Watabe et al. 2019).

Beyond its application as a free radionuclide for NIS-mediated uptake, ^211^At has also been extensively explored in the form of radiolabeled targeting constructs. When conjugated to targeting molecules, ^211^At demonstrates potent tumor-targeting and tumor-killing effects, which are driving clinical interest for refractory disease (Munekane et al. 2024). For example, [^211^At]PSAt-3-Ga has been developed for prostate cancer (El Fakiri et al. 2024), and ^211^At-APBA-Ga-DOTA-Kc(RGDfK) for glioblastoma (Echigo et al. 2024). Importantly, recent first-in-human SPECT/CT imaging of [^211^At]PSMA-5 further demonstrated highly specific tumor uptake in patients with metastatic prostate cancer, with biodistribution patterns closely matching PSMA PET tracers, thereby providing clinical proof-of-concept for targeted ^211^At radiopharmaceuticals (Watabe et al. 2025). In parallel, the successful scale-up production of [^211^At]PSMA-5 under investigational GMP conditions has recently been reported, enabling reproducible automated synthesis with high radiochemical purity and consistent batch-to-batch quality suitable for clinical administration (Naka et al. 2025). This work establishes the practical feasibility of translating ^211^At-based PSMA-targeted agents into investigator-initiated clinical trials. Moreover, PSMA-targeted ^211^At radiopharmaceuticals with high in vivo stability and favorable pharmacokinetics have been described previously (Mease et al. 2022), demonstrating that appropriate molecular design and optimized radiochemistry can effectively stabilize astatine in vivo.

However, the clinical translation of ^211^At-based radiopharmaceuticals is complicated by a chemical challenge: the inherent in vivo instability of At-labeled compounds (Burns et al. 2020; Kato et al. 2021; Yaginuma et al. 2025). This instability leads to delabeling and the release of free ^211^At^−^ (Gao et al. 2024; Yssartier et al. 2024), resulting in pronounced non-target redistribution within the body (Watabe et al. 2019). While pivotal for TAT, the in vivo chemistry of free astatine species remains poorly understood. Models extrapolated from iodide fail to predict its biodistribution, making the deciphering of its fundamental chemistry a prerequisite for radiopharmaceutical design (Oddstig et al. 2005; Spetz et al. 2013).

As the sole physiologically established pathway for active halide transport, the sodium-iodide symporter (NIS) represents the primary determining factor governing the in vivo behaviour of astatine. The NIS mediates the in vivo transport of astatine, along with a set of other anions such as the therapeutic radionuclides ^131^I^−^ and ^188^ReO_4_^−^, and the diagnostic agent ^99m^TcO_4_^−^ (Spetz et al. 2013; Watabe et al. 2019). This common uptake mechanism leads to accumulation of both ^131^I and ^211^At in NIS-expressing tissues such as the thyroid and gastric mucosa (Josefsson and Forssell-Aronsson 2012).

However, increasing experimental evidence indicates that the in vivo distribution of free ^211^At cannot be fully explained by NIS-mediated transport alone (Spetz et al. 2013), as substantial accumulation is also observed in non-NIS-expressing organs. The chemical origin of this divergence remains poorly understood; consequently, the chemical identity, transformation pathways, and organ-specific fate of free ^211^At under physiological conditions are not defined. Importantly, most previous studies have focused on biological transport pathways, while systematic investigations into the in vivo chemical speciation and transformation of free ^211^At remain scarce. As a result, the chemical processes underlying its distinct biodistribution have not been clearly elucidated. To address this fundamental knowledge gap, a combination of comparative biodistribution studies, experimental speciation analysis, and theoretical Pourbaix modelling is integrated in the present study to elucidate the radiochemical mechanisms governing the in vivo behaviour of free ^211^At.

## Methods

### Radionuclide production and preparation

^211^At was produced via the ^209^Bi(α,2n)^211^At nuclear reaction by irradiating a natural bismuth target with a 28 MeV α-particle beam using the CS-30 medical cyclotron at the Institute of Nuclear Science and Technology, Sichuan University. After dry distillation, the isolated radionuclide was dissolved in 0.1 M hydrochloric acid (HCl) to obtain an acidic ^211^At stock solution. (Naka et al. 2024).

Sodium iodide (^131^I) was purchased from Shanghai Atom Kexing Pharmaceuticals Co., Ltd. (Shanghai, China). Both radionuclides were diluted to their working concentrations immediately prior to use with sterile 0.9% saline (Baxter, USA) unless otherwise specified. Radiochemical purity (RCP > 98%) was confirmed by radio-thin layer chromatography (radio-TLC) using iTLC-SG strips (Agilent Technologies, USA) and a γ-counter (PerkinElmer Wizard^2^ 2470 Automatic Gamma Counter, USA) readout before all experiments. All radioactive solutions were freshly prepared prior to in vivo and in vitro studies. No major oxidized species were detected above the detection limit under the applied radio-TLC conditions.

### Animals and ethical approval

Female BALB/c mice (6–8 weeks old, 18–22 g) were obtained from Sibeifu (Suzhou) Laboratory Animal Co., Ltd. Animals were housed under standard specific pathogen-free (SPF) conditions (22 ± 2 °C, 50–60% humidity, 12 h light/dark cycle) with free access to food and water.

All animal experiments were conducted in accordance with institutional and national guidelines and were approved by the Ethics Committee of Shanghai Pudong Hospital, Fudan University (Approval No. 2025-D-Q-028).

### Ascorbic acid co-administration

Ascorbic acid (AA; sodium ascorbate) was freshly prepared in phosphate-buffered saline (PBS) immediately prior to use at a concentration of 20 mg/mL. AA was used as a chemical redox modulator in selected experiments.

For AA pretreatment, mice received an intraperitoneal injection of AA (100 µL per mouse). After an interval of 10–30 min, co-administration was performed.

For co-administration, Na^211^At was mixed with AA to obtain a homogeneous solution and injected promptly to minimize ex vivo alteration of astatine species. Under these conditions, AA was present in a large molar excess relative to ^211^At (AA: At ≥ 10^6^:1), consistent with its role as a redox-modulating agent. The final formulation exhibited a pH within the physiological range (pH ~ 6.8–7.4).

The injected activity of ^211^At and the total injection volume (100 µL) were identical to those used for the free Na^211^At group.

For comparative imaging controls, the same AA pretreatment protocol was also applied to selected ^131^I studies. In these experiments, mice received intraperitoneal AA pretreatment (100 µL, 20 mg/mL), followed 10–30 min later by intravenous administration of Na^131^I formulated with AA under the same total injection volume used for the free ^131^I group.

### Micro-SPECT/CT imaging

Whole-body single-photon emission computed tomography/computed tomography (SPECT/CT) scans were performed​ using a U-SPECT/CT system (MILabs, Utrecht, The Netherlands) for ^211^At studies, while a dedicated small-animal molecular imaging platform (Molecubes, Belgium) was used for ^131^I imaging.

Mice were anesthetized with 2.5% isoflurane (RWD Life Science, China) until loss of the pedal reflex, followed by maintenance at 1.5–2.0% during scanning. Animals were positioned in a standardized mouse holder with the head secured in a nose cone for continuous anesthetic delivery, and the body was gently immobilized to minimize motion artifacts.

For all imaging experiments, radiotracers were administered via tail-vein injection in awake mice without prior anesthesia. After injection, animals were returned to their cages and allowed to remain freely moving until the scheduled imaging time point (2 h post-injection). Anesthesia was induced immediately before SPECT/CT acquisition and maintained exclusively throughout the imaging procedure.

After intravenous tail-vein injection of radiotracers, whole-body SPECT/CT scans were acquired at 2 h post-injection. Imaging groups included free Na^131^I, Na^131^I with AA pretreatment/co-administration, free Na^211^At, and Na^211^At with AA pretreatment/co-administration. Injected activities were 100 µCi for Na^131^I studies and 50 µCi for Na^211^At studies unless otherwise specified, with a total injection volume of 100 µL. Image reconstruction was performed using a vendor-supplied ordered subset expectation maximization in 3D (OSEM-3D) algorithm (iterations = 6, subsets = 16) with attenuation and scatter correction for ^211^At datasets. Image visualization and analysis were carried out using VivoQuant software (version 4.5, inviCRO, USA).

### Biodistribution studies

For biodistribution analysis, mice received intravenous injections of Na^211^At or Na^131^I. At predetermined time points (10 min, 30 min, 2 h, 7 h, 20 h, and 44 h post-injection; *n* = 3 mice per time point), animals were euthanized and organs including the submandibular gland, pancreas, small intestine, large intestine, brain, heart, liver, spleen, lungs, kidneys, stomach, and thyroid were harvested.

Organs were weighed and their radioactivity quantified using a γ-counter (PerkinElmer Wizard^2^ 2470 Automatic Gamma Counter, USA), with a counting time of 30s per sample. Thyroid uptake was expressed as percentage of injected dose (%ID) per whole organ due to its extremely small mass, while other organs were expressed as percentage of injected dose per gram (%ID/g). All measurements were decay-corrected to the time of injection.

### In vitro serum incubation and speciation analysis

To investigate astatine transformation in protein-rich environments, 0.5 µCi of Na^211^At was incubated in 1 mL fetal bovine serum (FBS) at 37 °C for up to 14 h. Samples were analyzed by radio-thin layer chromatography (radio-TLC) using iTLC-SG glass fiber strips (Agilent Technologies, USA), consisting of silica gel (SG) impregnated glass fiber without binder. Physiological saline (0.9% NaCl) was used as the mobile phase.

Sequential anion-exchange chromatography using quaternary methyl ammonium (QMA) cartridges (Waters, USA) and C18 solid-phase extraction cartridges (Waters, USA) was employed to differentiate astatine species. Prior to use, the QMA cartridge​ was conditioned with 10 mL of 0.5 M NaHCO₃ followed by 10 mL of pure water. The C18 cartridge​ was conditioned with 10 mL of methanol followed by 10 mL of pure water. Each conditioning step was followed by brief air purging to remove residual liquid.

After incubation, the FBS sample was applied directly to the pre-conditioned QMA cartridge by gentle syringe push. The cartridge effluent (flow-through) was collected quantitatively as the “QMA filtrate” fraction. The QMA cartridge was then rinsed with 5 mL of pure water, and the rinse was combined with the initial flow-through. Radioactivity retained on the QMA cartridge was measured and assigned as the anionic fraction (QMA-retained, At^−^-enriched), whereas the combined flow-through/rinse was considered the non-anionic fraction (neutral/cationic and hydrophobic species).

The combined QMA flow-through fraction was subsequently loaded onto the pre-conditioned C18 cartridge. The C18 flow-through was collected as the hydrophilic fraction. The C18 cartridge was rinsed with 5 mL of pure water (combined with the flow-through), and the retained activity on the C18 cartridge was measured as the hydrophobic fraction (C18-retained, protein-associated/lipophilic species). Radioactivity in each fraction (QMA-retained, C18-retained, and final filtrate) was quantified using a γ-counter (PerkinElmer Wizard^2^ 2470). All activities were decay-corrected to the reference time point and expressed as percentage of total recovered activity.

As an iodide control, Na^131^I was incubated under matched conditions in FBS or PBS at 37 °C for 20 h and analyzed by radio-TLC using the same iTLC-SG/saline system.

### Ultrafiltration assay for colloidal ^211^At

Colloidal formation of ^211^At under physiological conditions was evaluated by incubating 0.5 µCi Na^211^At in 1 mL PBS at 37 °C for 15 h. Prior to use, 2 kDa ultrafiltration devices (Millipore, USA) were pre-rinsed with deionized water by centrifugation.

Samples were centrifuged at 12,000 rpm (approximately 13,000 × g) for 10 min, and radioactivity in both the filtrate and membrane-retained fractions was quantified using a γ-counter (PerkinElmer Wizard^2^ 2470 Automatic Gamma Counter, USA).

### Oxidation studies under simulated gastric and physiological conditions

For simulated gastric oxidation, Na^211^At was incubated in 0.1 M HCl, pepsin solution (1 mg/mL, Sigma-Aldrich), or combined HCl/pepsin at 37 °C. Aliquots were collected at defined time points (1, 2, 10, 12, and 14 h) and analyzed by radio-TLC on iTLC-SG strips using 0.9% saline as the mobile phase. As an iodide control, Na^131^I was incubated under the same simulated gastric conditions, including HCl, pepsin, and combined HCl/pepsin, at 37 °C. Aliquots were collected at 0, 1, 2, 10, 12, and 14 h and analyzed by radio-TLC on iTLC-SG strips using 0.9% saline as the mobile phase.

Oxidation kinetics under physiological oxidants were assessed by incubating 1 µCi of ^211^At in FBS at 37 °C in the presence of H_2_O_2_ (500 µM) or Fe^3+^ (100 µM), with aliquots analyzed by radio-TLC at 0.5, 1, 2, and 4 h.

### Toxicity evaluation

For toxicity assessment, mice injected with 30 µCi Na^211^At were evaluated at acute (day 3) and subacute (day 15) time points. Blood samples were collected for serum biochemical analysis of alanine aminotransferase (ALT), aspartate aminotransferase (AST), alkaline phosphatase (ALP), creatinine, and urea using an automated biochemical analyzer.

Major organs (small intestine, liver, spleen, thyroid) were collected, fixed in 4% paraformaldehyde, paraffin-embedded, and processed for hematoxylin and eosin (H&E) histological examination.

### Statistical analysis

Data analysis and plotting were performed using Microsoft Excel 2019, OriginPro 2024b (OriginLab, USA), and GraphPad Prism 9.0 (GraphPad Software, USA). Quantitative results are presented as mean ± standard deviation (SD).

For radio-TLC analysis, developed strips were segmented into ten equal sections, each quantified individually by γ-counting. Chromatographic profiles were reconstructed and smoothed using the Savitzky-Golay algorithm for visualization. No formal hypothesis testing was performed unless otherwise stated.

### Computational methods and Pourbaix analysis

Quantum-chemical calculations were performed using Gaussian 16 (Frisch et al. 2016). Thermochemical corrections were applied at 310.15 K and 1 bar. Solvation effects were modeled using the SMD implicit solvent model (water). Gibbs free energies were used to construct Pourbaix diagrams and evaluate redox-dependent astatine speciation under physiological conditions.

To account for the heavy-element nature of astatine, relativistic effects were explicitly considered in the quantum-chemical calculations. Geometry optimizations were performed at the B3LYP level, while single-point energies were refined using a spin-orbit density functional theory (SODFT) approach based on the gB3LYP formalism (Champion et al. 2010). A relativistic effective core potential (ECP60MDF) was employed for astatine in combination with appropriate valence basis sets, enabling the inclusion of both scalar relativistic and spin-orbit coupling effects. These relativistically corrected Gibbs free energies were subsequently used for the construction of Pourbaix diagrams and the evaluation of redox-dependent astatine speciation under physiological conditions. For clarity, throughout the main text, astatine species are described using formal oxidation-state notation (e.g., At^−^, At^+^, At^3+^) to reflect their dominant redox character in biological environments. In the computational analysis (ESI), these species are represented by their corresponding oxide or hydroxo complexes (e.g., AtO^−^, AtO_2_^−^), which serve as thermodynamic proxies for the experimentally relevant oxidation states.

## Results

### Comparative in vivo distribution of free ^211^At and ^131^I

**Fig. 1:**
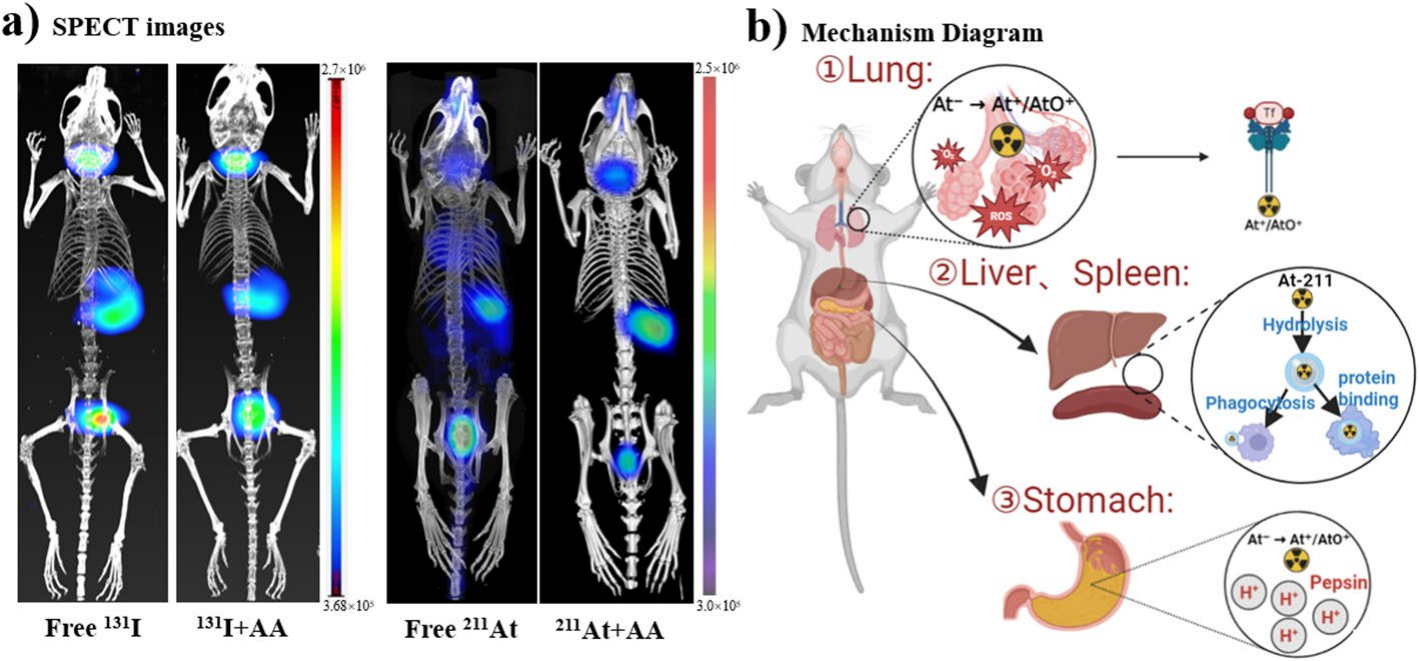
In vivo distribution and proposed mechanisms of ^211^At organ accumulation. **a** Representative whole-body micro-SPECT/CT images of BALB/c mice (*n* = 3 per group) acquired at 2 h after intravenous administration of free ^131^I (100 µCi), ^131^I + ascorbic acid (^131^I + AA), free ^211^At (50 µCi), and ^211^At + ascorbic acid (^211^At + AA), arranged from left to right. Images are displayed using radionuclide-specific intensity scales (Bq/mL), as indicated by the corresponding colour bars. Because ^131^I and ^211^At were acquired on different SPECT systems, signal intensities are not intended for direct quantitative comparison between radionuclides. **b** Schematic illustrating the proposed organ-specific mechanisms of off-target ^211^At deposition, including​ reactive oxygen species (ROS)-driven oxidation to cationic species (e.g., AtO^+^) in the lungs; hydrolysis and colloidal formation in the liver and spleen; and acidic hydrolysis to At^+^ in the stomach

Whole-body micro-SPECT/CT imaging at 2 h post-injection demonstrated that free ^131^I and free ^211^At exhibited a broadly similar initial distribution pattern (Fig. 1a). Both radionuclides showed prominent uptake in NIS-expressing tissues, including the thyroid and stomach, consistent with active halide transport.

However, clear organ-specific differences emerged upon further analysis. Quantitative biodistribution studies revealed that free ^211^At accumulated markedly in several non-target organs, including the stomach, lungs, liver, and spleen, whereas free ^131^I exhibited minimal uptake outside thyroidal tissues (Fig. S1, ESI). In particular, gastric uptake of ^211^At was substantially higher than that of ^131^I and persisted over extended time points. Similarly, lungs and hepatosplenic retention of ^211^At was markedly greater than that observed for iodide. For comparative imaging controls, an additional ^131^I + AA group was also included in Fig. 1a. In contrast to ^211^At, co-administration of AA did not visibly alter the overall distribution pattern of ^131^I, consistent with the higher redox stability of iodide under physiological conditions.

**Fig. 2:**
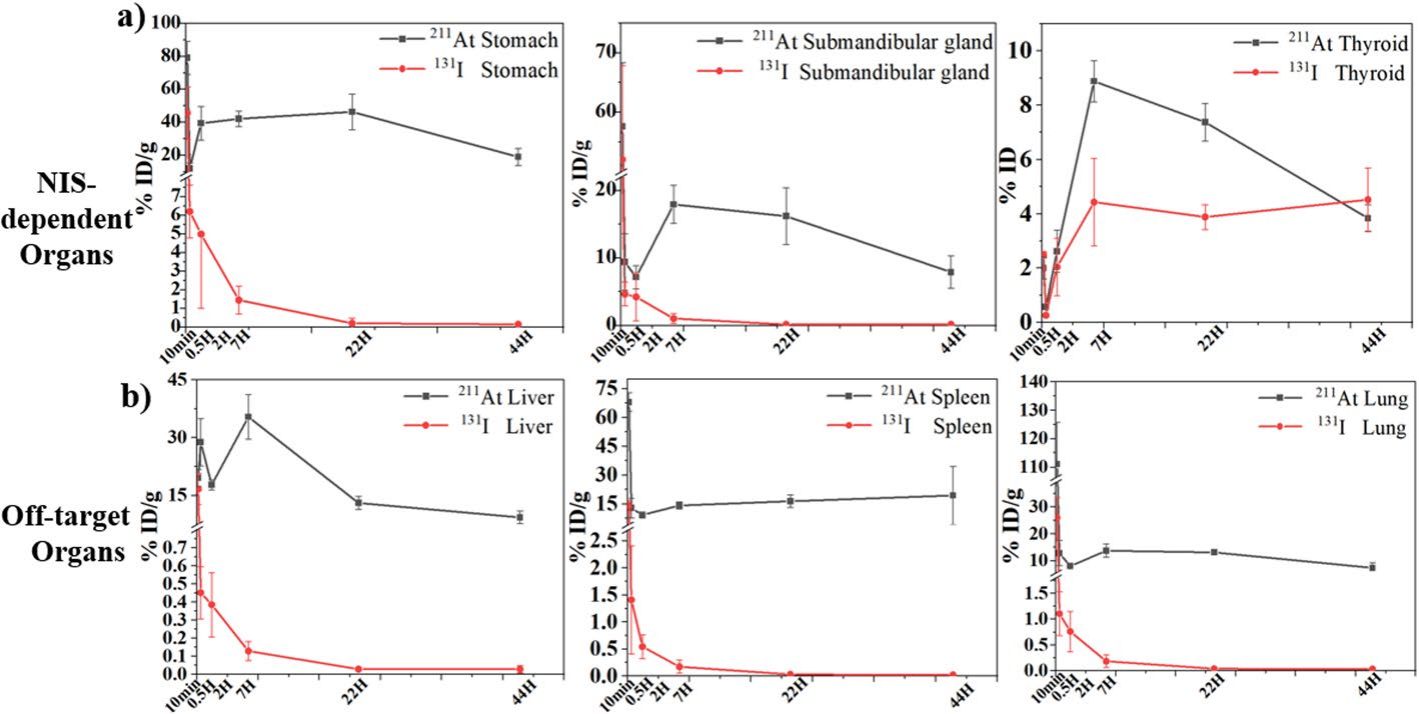
Biodistribution kinetics of free ^211^At and ^131^I in major organs. **a** Time-dependent uptake of ^211^At and ^131^I in NIS-expressing tissues, including the stomach, submandibular gland, and thyroid. **b** Uptake profiles of ^211^At and ^131^I in representative non-target organs, including the liver, spleen, and lungs. Data are shown as mean ± SD (*n* = 3 per time point) for each radionuclide

Longitudinal biodistribution analysis confirmed that this off-target accumulation of ^211^At was not transient. While early uptake (0.5 h post-injection) in NIS-positive tissues was comparable between the two radionuclides, ^211^At showed prolonged retention thereafter, particularly in the stomach and submandibular glands, exceeding that of ^131^I by several fold (Fig. 2a). In contrast, ^131^I exhibited progressive clearance from these tissues over time. In non-NIS organs, sustained accumulation of ^211^At was observed up to 44 h post-injection (Fig. 2b), indicating a different pharmacokinetic profile compared with iodide.

To rationalize the observed divergence in organ accumulation between ^211^At and ^131^I, a schematic overview of the proposed chemical transformation pathways of free ^211^At in vivo is presented in Fig. 1b. This diagram summarizes the redox-driven processes inferred from imaging, biodistribution, and speciation experiments, highlighting oxidation to cationic species in oxidative environments (e.g., lungs), acid-promoted transformation in the stomach, and subsequent hydrolysis and colloidal formation leading to hepatosplenic retention.

### Effect of ascorbic acid on the in vivo distribution of ^211^At

To examine whether the abnormal biodistribution of ^211^At is associated with redox-driven chemical transformation, AA was co-administered with free ^211^At as a reducing agent. Whole-body and transverse micro-SPECT/CT images showed that AA co-injection visibly reduced radioactive signals in several non-target organs, particularly the stomach and lungs (Fig. 1a and Fig. S2, ESI).

Compared with free ^211^At alone, the ^211^At + AA group exhibited an overall redistribution pattern closer to that of ^131^I, with reduced extra-thyroidal accumulation. By contrast, the corresponding ^131^I + AA control did not show an obvious redistribution relative to free ^131^I in the whole-body SPECT images (Fig. 1a), supporting that the effect of AA is more closely related to suppression of redox-sensitive astatine transformation rather than a nonspecific imaging artifact. These imaging results indicate that antioxidant treatment can partially suppress the formation of chemically reactive astatine species responsible for off-target deposition, supporting a redox-dependent mechanism governing the in vivo distribution of free ^211^At.

### Oxidation and speciation of ^211^At in biological environments

**Fig. 3:**
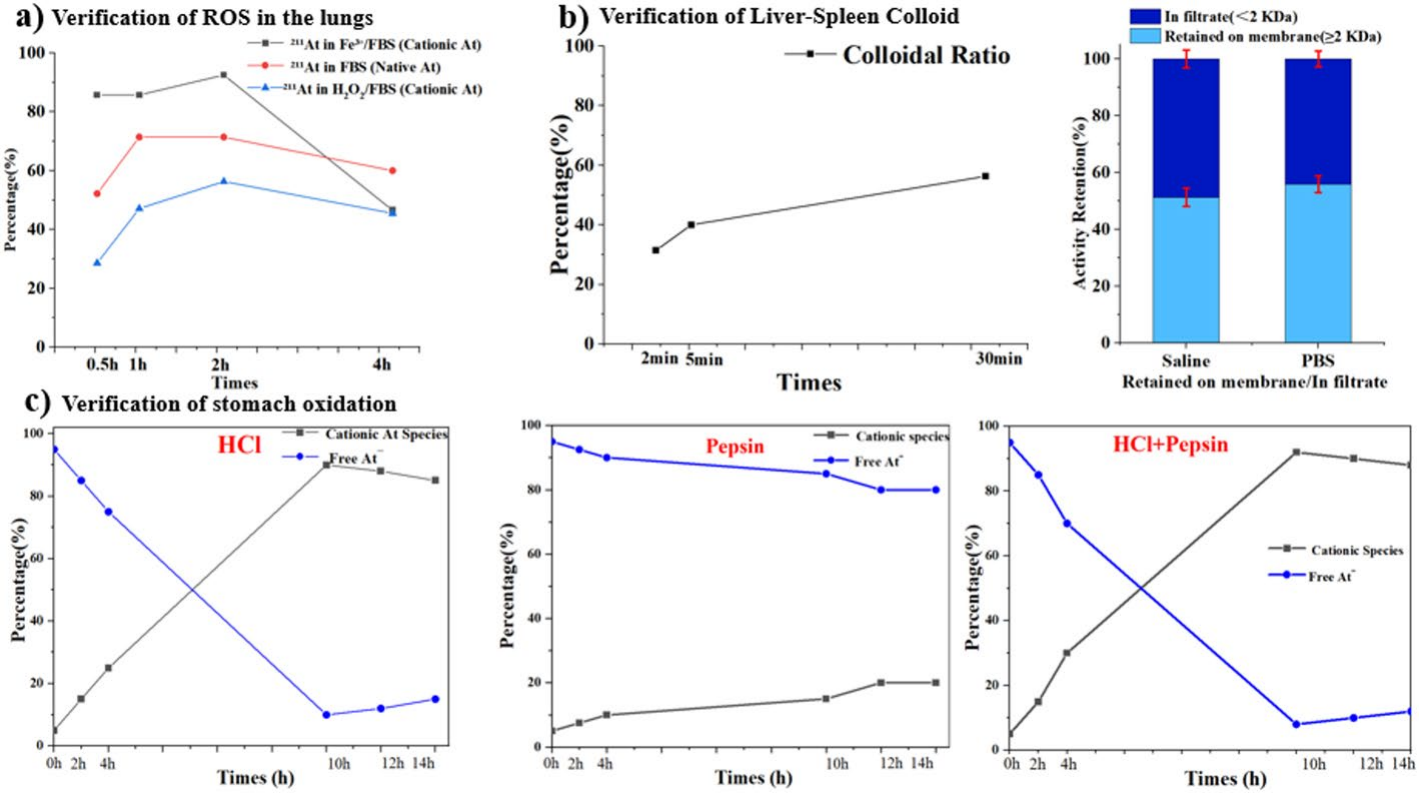
Mechanistic pathways of ^211^At speciation and deposition. **a** Time-dependent oxidation kinetics of ^211^At induced by strong (H_2_O_2_) and mild (Fe^3+^) oxidants. **b** Comparative stability and colloidal formation ratio of ^211^At in physiological (PBS) versus native solutions; colloidal formation rate in physiological media. **c** Speciation profile of ^211^At under gastric conditions (HCl, Pepsin), illustrating the formation of cationic species

To directly investigate the chemical transformations of ^211^At under physiologically relevant conditions, in vitro oxidation and speciation studies were performed. Incubation of ^211^At^−^ in FBS led to the rapid appearance of non-anionic astatine species, as evidenced by radio-TLC analysis (Fig. 3a). These species were absent in freshly prepared ^211^At stock solutions, indicating oxidative conversion in protein-rich environments (Table S1, ESI). Under matched incubation conditions, iodide (^131^I) remained predominantly at the solvent front in both FBS and PBS without evidence of less mobile or non-anionic species (Fig. S7, ESI), indicating that the serum-induced transformation observed for ^211^At is not a general feature of halide radionuclides.

Further experiments using defined oxidants demonstrated that strong oxidants (H_2_O_2_) and mild oxidants (Fe^3+^) induced time-dependent oxidation of ^211^At^−^ to cationic species (Fig. 3a and Fig. S3, ESI). These findings are consistent with the presence of reactive oxygen species and redox-active metal ions in vivo.

### Colloidal formation and hepatosplenic retention of oxidized ^211^At

Ultrafiltration experiments revealed that a substantial fraction of ^211^At activity was retained by a 2 kDa molecular weight cutoff membrane following incubation in physiological media (PBS or saline), with retention ratios of 51.28% and 55.87%, respectively (Fig. 3b). This observation is consistent with the formation of colloidal or macromolecular astatine-containing species (Fig. S4, ESI), although the precise structural nature of these species cannot be conclusively resolved by ultrafiltration alone. Consistent with this interpretation, iodide did not show evidence of less mobile or colloid-like species after prolonged incubation in PBS or FBS by radio-TLC (Fig. S7, ESI), further indicating that the formation of retained macromolecular/colloidal fractions is a characteristic feature of astatine chemistry.

The formation of such colloidal products provides a chemical basis for the pronounced liver and spleen accumulation observed in vivo. These larger aggregates are likely cleared by the reticuloendothelial system, particularly macrophages, resulting in sustained hepatosplenic retention.

### Acid-induced transformation of ^211^At under gastric conditions

To simulate the gastric microenvironment, ^211^At was incubated in hydrochloric acid (HCl) with or without pepsin. Radio-TLC analysis revealed a progressive increase in cationic astatine species under acidic conditions, reaching approximately 90% after 10 h (Fig. S5, ESI). The presence of pepsin did not markedly alter the speciation profile, indicating that enzymatic activity plays a minimal role in astatine transformation in the stomach.

These results suggest that the highly acidic gastric environment lowers the redox threshold for oxidation of ^211^At^−^, promoting conversion to neutral or cationic species with increased membrane permeability, thereby contributing to gastric deposition observed *in vivo.* Under the same simulated gastric conditions, iodide (^131^I) remained predominantly in the anionic form across all tested time points and media (HCl, pepsin, and HCl/pepsin), without detectable oxidation or formation of non-anionic species by radio-TLC (Fig. S6, ESI). This contrast further supports that the acid-promoted transformation observed in the stomach is a specific feature of astatine rather than a general property of halide radionuclides.

### Theoretical Pourbaix analysis of astatine redox behaviour

**Fig. 4:**
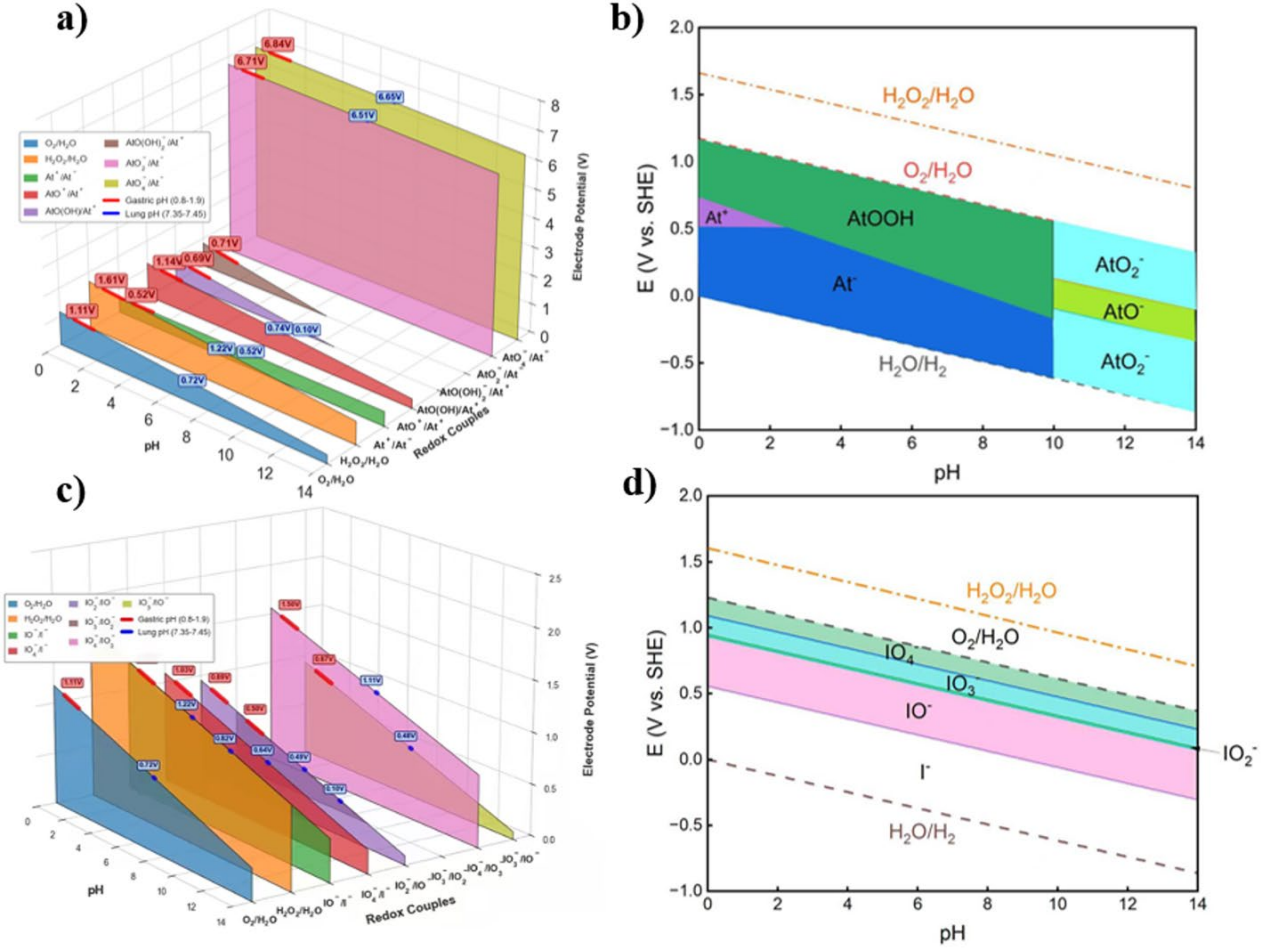
Theoretical redox analysis of astatine and iodide under physiological conditions. **a** Calculated redox-couple potentials of ^211^At species as a function of pH and electrode potential (E, vs. the standard hydrogen electrode, SHE), with physiological gastric and lung pH ranges indicated. **b** Calculated Pourbaix predominance diagram of ^211^At, showing the stability domains of At^−^, At^+^, AtOOH, AtO^−^, and AtO_2_^−^ under aqueous conditions. **c** Calculated redox-couple potentials of iodide species under the same theoretical framework, with physiological gastric and lung pH ranges indicated. **d** Calculated Pourbaix predominance diagram of iodide, showing the stability domains of I^−^, IO^−^, IO_3_^−^, IO_4_^−^, and IO_2_^−^ under aqueous conditions. Together, these calculations highlight the markedly greater redox sensitivity of astatine relative to iodide and provide a theoretical basis for their distinct in vivo chemical behaviors

Because astatine lacks stable isotopes and experimental redox data are limited, density functional theory (DFT)-based electrochemical calculations were performed to evaluate its redox behaviour under physiological conditions. The Pourbaix diagram (Fig. 4), constructed based on the calculated solvation free energies (Table S2, ESI),​ demonstrates that astatine speciation is strongly governed by pH and electrochemical potential.

Within the physiological pH range (6.8–7.4), At^−^ is predicted to be thermodynamically unstable and prone to oxidation even in the presence of mild biological oxidants. Fe^3+^ is predicted to oxidise At^−^ to At^+^, while O_2_ and H_2_O_2_ can further oxidise At^−^ to At^3+^.

At^3+^ (At^3+^ is used here to denote a formal oxidation state representing highly oxidized astatine species rather than a discrete, isolable molecular ion) is identified as a metastable intermediate that rapidly undergoes hydrolysis near neutral pH to form colloidal AtOOH, which is predicted to be the thermodynamically preferred form in the liver and spleen. Importantly, oxidation to higher formal oxidation states (+ 5 or + 7) is not thermodynamically favored within the physiologically relevant Eh-pH window, constraining biologically relevant astatine chemistry to At^−^, At^+^, At^3+^, and colloidal hydrolysis products. Although higher oxidation states were included in the computational model for completeness, their stability domains lie outside the redox conditions expected in vivo.

### Preliminary evaluation of in vivo toxicity

**Fig. 5:**
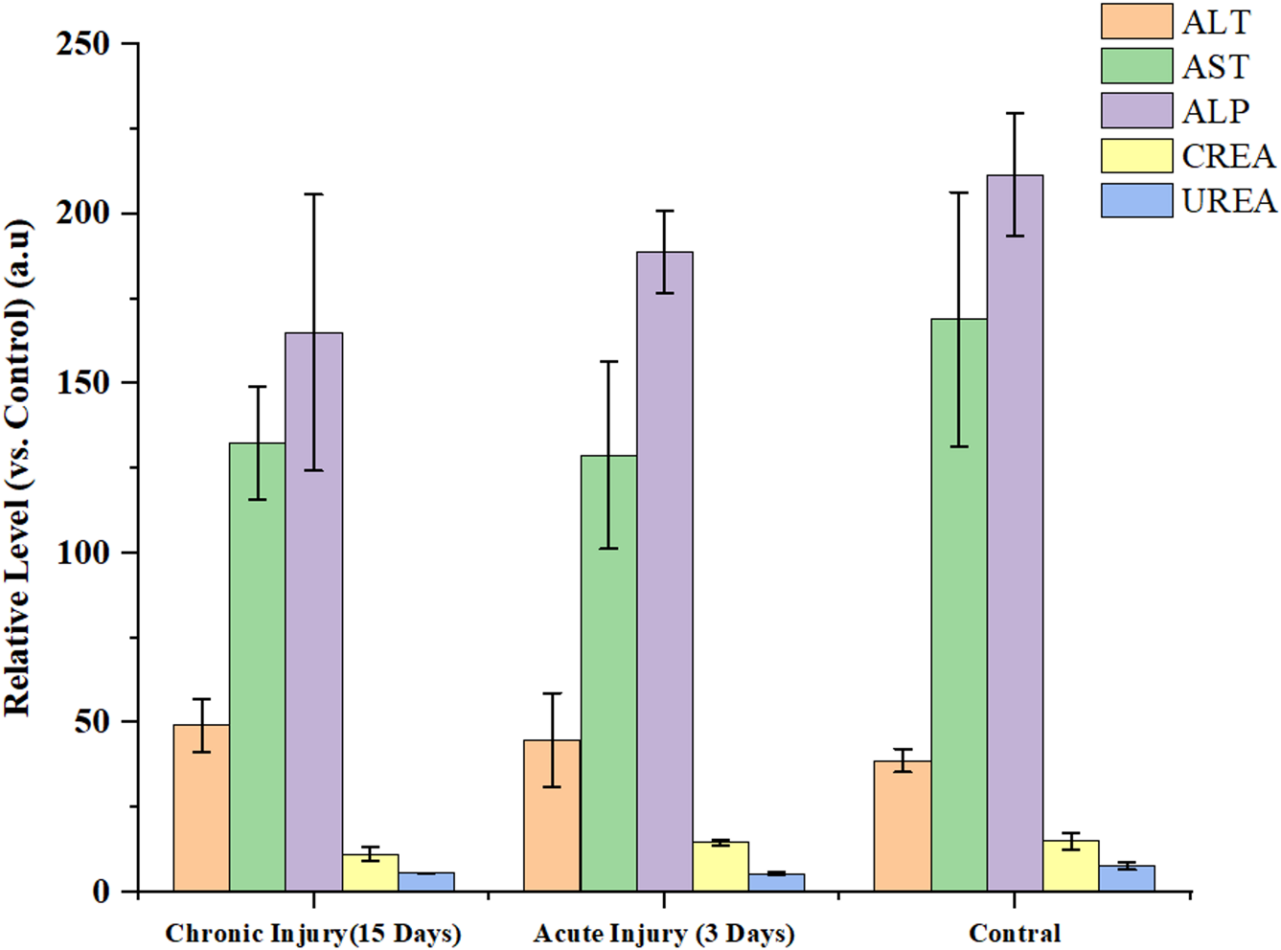
Relative serum levels of hepatic and renal function markers. Normalized serum levels (relative to healthy controls) of the indicated biomarkers (ALT, AST, ALP, creatinine, urea) are shown for the acute (day 3) and subacute (day 15) phases

**Fig. 6:**
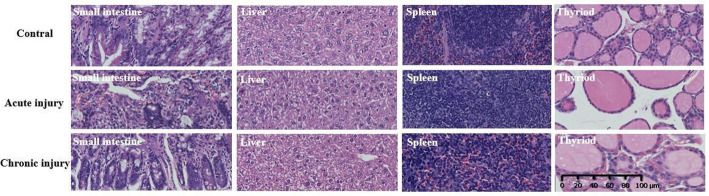
Representative H&E-stained sections of major organs following acute and subacute exposure to Na^211^At. Microscopic images of the small intestine, liver, spleen, and thyroid collected from control, acute-injury (day 3), and chronic-injury (day 15) groups show no overt radiation-induced structural damage or inflammatory lesions. Tissue morphology remained comparable to that of untreated controls

Finally, preliminary biosafety assessments were conducted to evaluate potential non-target toxicity associated with free ^211^At. Serum biochemical analysis and histopathological examination of major organs revealed no significant hepatic or renal dysfunction at administered activities up to 30 µCi per mouse (Figs. 5 and 6). Despite the observed off-target accumulation, histological damage remained limited (Table S3, ESI), consistent with the short tissue penetration range of α-particles.

## Discussion

The present study systematically demonstrates that the distinctive in vivo biodistribution of free ^211^At arises from its intrinsic redox instability rather than from NIS-mediated transport alone. Although ^211^At and ^131^I initially share a broadly similar whole-body distribution pattern, their subsequent pharmacokinetic behaviors diverge markedly, particularly in non-NIS-expressing organs such as the lungs, liver, spleen, and stomach. This divergence arises from the distinct chemistry of astatine: its pronounced metalloid character and highly polarizable electron cloud make At^−^ highly susceptible to oxidation under physiological conditions, yielding cationic species (e.g., At^+^, At^3+^) with unique in vivo behavior (Burns et al. 2020; Spetz et al. 2013b). This interpretation is further supported by matched iodide control experiments, which showed no appreciable oxidation or formation of less mobile species under either simulated gastric or serum/PBS incubation conditions (Figs. S6 and S7, ESI).

SPECT imaging and quantitative biodistribution analyses consistently showed pronounced and persistent accumulation of ^211^At in these organs, whereas ^131^I exhibited rapid clearance outside the thyroid. This divergence cannot be fully explained by classical halide transport mechanisms and instead reflects fundamental differences in the physicochemical properties of astatine relative to iodine (Guérard et al. 2021).

Ex vivo serum incubation, radio-TLC, ion-exchange chromatography, and ultrafiltration experiments collectively reveal a sequential transformation pathway for free ^211^At under physiologically relevant conditions. It should be noted that FBS was employed as a protein-rich medium to model oxidative and binding environments in circulation. FBS is widely used as a standardized biological matrix due to its high protein content and reproducible redox properties, allowing mechanistic evaluation of astatine speciation processes. While species-specific differences between bovine and murine serum may exist, the present study focuses on fundamental chemical transformation pathways rather than absolute quantitative kinetics. Future investigations using mouse serum could further refine physiological relevance; however, the consistent correlation between in vitro speciation trends and in vivo biodistribution observed here supports the validity of FBS as an effective mechanistic proxy. Initially injected as At^−^, astatine rapidly undergoes oxidation to neutral or cationic species in protein-rich and oxidizing environments (Guérard et al. 2021). These oxidized species exhibit altered chromatographic behavior and reduced anion-exchange affinity, consistent with the formation of At^+^- or AtO^+^-type intermediates. Notably, under matched incubation conditions, iodide remained stable in both FBS and PBS without detectable formation of less mobile or non-anionic species (Fig. S7, ESI), supporting that the serum-induced transformation observed here is specific to astatine chemistry rather than a nonspecific artifact of the protein matrix.

Subsequently, these cationic species undergo hydrolysis near neutral pH to generate colloidal or macromolecular aggregates (Champion et al. 2013), as evidenced by significant retention on 2 kDa ultrafiltration membranes. It should be noted that retention in ultrafiltration assays does not exclusively indicate the presence of inorganic colloids. Protein-associated aggregates or other macromolecular complexes involving oxidized astatine species may also contribute to the observed retention. The formation of such colloidal species provides a mechanistic explanation for the pronounced hepatic and splenic retention of ^211^At via the reticuloendothelial system, a behavior not observed for iodide. Consistent with this interpretation, iodide did not show detectable formation of less mobile species after prolonged incubation in FBS or PBS by radio-TLC (Fig. S7, ESI), supporting the view that the retained macromolecular/colloid-like fractions observed in this study are more closely associated with oxidized astatine chemistry.

The observed organ-specific accumulation patterns can be rationalized by considering local biochemical microenvironments. The lungs, which are known to present a relatively oxidative microenvironment, provide favorable conditions for oxidative conversion of At^−^ to cationic forms, promoting nonspecific protein binding and tissue retention (Scudiero et al. 2008). In the liver and spleen, subsequent hydrolysis of these oxidized species leads to colloidal formation and macrophage-mediated sequestration.

In the stomach, the highly acidic milieu substantially lowers the redox threshold for At^−^ oxidation (Zalutsky and Vaidyanathan 2000). Radio-TLC analysis under simulated gastric conditions confirmed rapid conversion of At^−^ to cationic species, independent of enzymatic activity. These species likely exhibit enhanced membrane permeability and chemical trapping within the gastric mucosa, explaining the exceptionally high gastric uptake observed in vivo. Under the same simulated gastric conditions, iodide remained predominantly anionic across HCl, pepsin, and HCl/pepsin media without detectable oxidation by radio-TLC (Fig. S6, ESI). This contrast indicates that the acid-promoted transformation observed in the stomach is not a general feature of halide radionuclides, but a distinct consequence of astatine redox chemistry.

Importantly, co-administration of AA partially suppressed abnormal organ accumulation of ^211^At, as demonstrated by SPECT imaging. It should be noted that ascorbic acid may influence astatine biodistribution through multiple mechanisms, including redox modulation, local pH buffering, and potential effects on physiological transport pathways. While the present results support a redox-sensitive component, further studies using pH-matched or non-redox-active controls would be required to fully disentangle these contributions. In contrast, the corresponding ^131^I + AA imaging control did not show an obvious redistribution relative to free ^131^I, supporting that the attenuation observed for ^211^At is more closely related to suppression of redox-sensitive astatine transformation than to a nonspecific effect of co-administered AA. This finding provides direct in vivo evidence that astatine speciation—and consequently its biodistribution—can be modulated by redox control. By stabilizing astatine in a reduced state, antioxidant treatment limits the formation of oxidized and hydrolyzed species responsible for off-target deposition (Watabe et al. 2019). This observation underscores the primacy of chemical mechanisms over purely biological processes​ in governing ^211^At biodistribution and highlights redox chemistry as a critical determinant of its in vivo fate.

DFT-derived Pourbaix analysis further supports the experimentally observed behavior. Under physiological pH and redox conditions, At^−^ is thermodynamically unstable and readily oxidized to At^+^ or At³^+^ by biologically relevant oxidants such as Fe^3+^, O_2_, and H_2_O_2_. At^3+^ is predicted to be metastable and to undergo spontaneous hydrolysis near neutral pH, yielding colloidal AtOOH-type or related hydrolyzed astatine species that are thermodynamically favored in hepatic and splenic environments.

Importantly, higher oxidation states (+ 5 or + 7) are not accessible under physiological redox potentials, constraining biologically relevant astatine chemistry to a limited set of reduced, cationic, and hydrolyzed forms. This theoretical framework provides a unified chemical explanation for the experimentally observed organ-specific accumulation patterns. It should be emphasized that the DFT-derived Pourbaix analysis provides a thermodynamic framework for assessing feasible redox pathways rather than a direct description of in vivo reaction kinetics. The experimental observations therefore constitute the primary evidence for astatine transformation, with the theoretical analysis serving to rationalize the observed trends.

Although acute and subacute toxicity assessments indicated acceptable hepatic and renal safety within the investigated activity range, the pronounced off-target accumulation of free ^211^At highlights a critical challenge for its clinical translation (Joho et al. 2025). The present findings highlight​ the importance of controlling astatine redox chemistry, which is essential​ for mitigating nonspecific deposition and improving the in vivo stability of ^211^At-based radiopharmaceuticals (Feng et al. 2023; Suzuki et al. 2025; Watabe et al. 2025b).

Overall, this work, together with matched iodide control experiments, establishes redox-driven speciation and colloid formation as dominant chemical processes that distinguish the in vivo chemistry of ^211^At from that of iodide. These insights provide a fundamental chemical basis for the rational design, stabilization, and purification of astatine radiopharmaceuticals, extending beyond empirical labeling strategies toward mechanism-guided radiochemistry.

## Conclusion

This study demonstrates that the in vivo chemistry of free astatine-211 is fundamentally distinct from that of iodide and is governed primarily by intrinsic redox instability. Combined experimental and computational analyses, supported by matched iodine-131 control studies, identify oxidation, hydrolysis, and colloid-like/macromolecular fraction formation as the key processes underlying its characteristic organ distribution. These findings provide a chemically grounded framework for understanding free astatine biodistribution and for guiding rational stabilization strategies in the development of astatine-based radiopharmaceuticals.

## Supplementary Information

Below is the link to the electronic supplementary material.

Supplementary Material 1.

Supplementary Material 2.

## Data Availability

All data supporting the findings of this study are available within the article and its supplementary information files (ESI).

## Abbreviations

AA: Ascorbic acid
ALT: Alanine aminotransferase
ALP: Alkaline phosphatase
AST: Aspartate aminotransferase
At: Astatine
DFT: Density functional theory
DSBs: DNA double-strand breaks
FBS: Fetal bovine serum
H&E: Hematoxylin and eosin
HCl: Hydrochloric acid
LET: Linear energy transfer
NIS: Sodium-iodide symporter
OSEM: Ordered subset expectation maximization
PBS: Phosphate-buffered saline
QMA: Quaternary methyl ammonium
RCP: Radiochemical purity
ROS: Reactive oxygen species
SHE: Standard hydrogen electrode
SPECT/CT: Single-photon emission computed tomography/computed tomography
SPF: Specific pathogen-free
TAT: Targeted alpha therapy
TLC: Thin-layer chromatography
