# Supplementary material for: Redox-driven speciation and colloid formation contribute the in vivo chemistry and organ deposition of Astatine-211
Source: EJNMMI Radiopharm Chem. 2026 Mar 31;11:34. doi: 10.1186/s41181-026-00441-3 (PMC13172141; doi:10.1186/s41181-026-00441-3)
Supplement: Supplementary file 1 — Supplementary Material 1. [file 41181_2026_441_MOESM1_ESM.docx]

Electronic Supplementary Information (ESI)

Electronic Supplementary Information (ESI) – Contents

S1. Materials and Experimental Methods

S1.1 Radionuclide Source and Preparation

S1.2 Micro-SPECT/CT imaging

S1.3 Biodistribution studies

S1.4 Serum incubation studies

S1.5 Ultrafiltration assay for colloidal ²¹¹At

S1.6 Simulated gastric fluid oxidation

S1.7 Oxidation kinetics under physiological oxidants

S1.8 Biosafety evaluation

S1.9 Statistical analysis

S2. Computational study of astatine and iodide redox behaviour and Pourbaix analysis

S2.1 Computational methods

S2.2 Construction and interpretation of the astatine and iodide Pourbaix diagram

S3. Supplementary Figures

Fig. S1 Comparative biodistribution and pharmacokinetics of Na²¹¹At and Na¹³¹I in BALB/c mice

Fig. S2 Transverse SPECT/CT images show the distribution of ²¹¹At in the liver, lung, stomach, and thyroid

Fig. S3 Time-dependent oxidation of ²¹¹At by H₂O₂ and Fe³⁺ analyzed by radio-thin layer chromatography (radio-TLC)

Fig. S4 Colloid formation and ultrafiltration (2 kDa cutoff)

Fig. S5 ²¹¹At oxidation kinetics under simulated gastric conditions

Fig. S6 ¹³¹I speciation under simulated gastric conditions

Fig. S7 Stability of ¹³¹I in FBS and PBS after 20 h incubation at 37 °C

S4. Supplementary Tables

Table S1 Sequential cartridge analysis of ²¹¹At species

Table S2 Calculated solvation free energies (Gₛₒₗᵥ) of astatine species at the B3LYP/SMD level (310 K)

Table S3 Calculated solvation free energies (G_solv_) of iodide-related species and reference molecules at the matched theoretical level (310 K)

Table S4 Serum biochemical parameters following ²¹¹At administration

Reference

S1. Materials and Experimental Methods

S1.1 Radionuclide Source and Preparation

The production, isolation, and formulation of ²¹¹At and ¹³¹I were carried out as described in the Methods section of the main text. Briefly, Na²¹¹At was supplied in acidic solution following dry distillation, while Na¹³¹I was commercially obtained. Working solutions were freshly prepared prior to each experiment.

Radiochemical purity of both radionuclides (>98%) was confirmed by radio-TLC using the same analytical conditions as reported in the main article.

S1.2 Micro-SPECT/CT imaging

Micro-SPECT/CT imaging was performed using the same systems and protocols described in the main Methods section. Supplementary acquisition and reconstruction parameters are provided here for completeness.

Reconstruction of ²¹¹At datasets was performed using OSEM-based iterative algorithms with attenuation and scatter correction. Image co-registration and quantitative analysis were conducted using VivoQuant software.

S1.3 Biodistribution studies

Biodistribution experiments were conducted following the procedures described in the main Methods section. Quantitative organ uptake values were calculated as %ID/g, except for thyroid uptake, which was expressed as %ID per organ. Complete biodistribution datasets are presented in Fig. S1 (ESI).

S1.4 Serum incubation studies

To support the *in vivo* observations, ²¹¹At was incubated in fetal bovine serum under physiological conditions. Time-dependent speciation changes were monitored by radio-TLC and further characterized using QMA anion-exchange cartridges and C18 solid-phase extraction.

These supplementary experiments provide mechanistic evidence for protein-associated and non-anionic astatine species discussed in the main text.

Additional iodide control experiments were performed to assess the stability of ¹³¹I under identical incubation conditions. Na¹³¹I (0.5 μCi) was incubated in 1 mL FBS or PBS at 37 °C for 20 h. Aliquots were subsequently analyzed by radio-TLC on iTLC-SG strips using 0.9% saline as the mobile phase. Chromatographic profiles were reconstructed and quantified in the same manner as described for ²¹¹At. No significant shift in Rf distribution or formation of less mobile species was observed for ¹³¹I in either FBS or PBS, indicating the absence of oxidative transformation or colloidal formation under these conditions (Fig. S7, ESI).

S1.5 Ultrafiltration assay for colloidal ²¹¹At

To assess colloidal formation under physiological conditions, 0.5 μCi of Na²¹¹At was added to 1 mL of pH 7.4 PBS and incubated at 37 °C for 15 h. Before use, 2 kDa ultrafiltration tubes were pre-rinsed with deionized water by centrifugation to remove potential contaminants. The incubated samples were then centrifuged through the membranes at 12,000 rpm, and radioactivity in both the filtrate and the retained fraction was quantified using a γ-counter. Substantial retention of activity on the membrane indicated the formation of colloidal or aggregated hydrolyzed ²¹¹At species, which is consistent with​ the proposed hydrolysis and colloid formation pathway.

S1.6 Simulated gastric fluid oxidation

To investigate gastric accumulation mechanisms, Na²¹¹At was incubated in hydrochloric acid, pepsin solution, or a combined HCl/pepsin mixture at 37 °C. Aliquots were withdrawn at 1, 2, 10, 12 and 14 h, and speciation was assessed by radio-TLC. For chromatographic confirmation, samples were further analyzed on QMA anion-exchange cartridges, which were pre-activated by rinsing with 10 mL of 0.5 M sodium bicarbonate prior to use. Time-dependent loss of the anionic At⁻ fraction and formation of neutral/cationic products confirmed rapid acid-promoted oxidation of ²¹¹At under simulated gastric conditions (Fig. S5, ESI). As an iodide control, Na¹³¹I was incubated under the same simulated gastric conditions (HCl, pepsin, and HCl/pepsin; 37 °C) and analysed by radio-TLC at matched time points. No detectable oxidation or formation of non-anionic species was observed (Fig. S6, ESI).

S1.7 Oxidation kinetics under physiological oxidants

The oxidation sensitivity of ²¹¹At was examined by incubating 1 μCi of ²¹¹At in a total volume of 100 μL FBS​ at 37 °C under three conditions: (1) H₂O₂, (2) Fe³⁺, (3) control (FBS only). Aliquots were collected at 0.5, 1, 2, and 4 h and analyzed on iTLC-SG (Fig. S3, ESI).

S1.8 Biosafety evaluation

To further investigate the biological effects within the effective therapeutic dose range (8.65–52.17 µCi),^1^ we employed a dose of 30 µCi of Na²¹¹At per mouse.

S1.9 Statistical analysis

All supplementary data were decay-corrected to the time of injection. Radio-TLC strips were segmented and quantified by γ-counting, and chromatographic profiles were reconstructed and smoothed using the Savitzky–Golay algorithm for visualization purposes only.

S2. Computational study of astatine redox behaviour and Pourbaix analysis

S2.1 Computational methods

All quantum-chemical calculations were performed using Gaussian 16. Thermochemical corrections were obtained at a temperature of 310.15 K and a pressure of 1 bar in order to approximate physiological conditions. All redox potentials were referenced to the standard hydrogen electrode (SHE), for which the potential is defined as 0 V at all temperatures.

Geometry optimisations and harmonic frequency calculations were carried out using the B3LYP density functional. For astatine-containing species, spin–orbit coupling effects were taken into account using the gB3LYP formalism (spin–orbit DFT, SODFT)^2–5^ for single-point energy calculations on the optimised geometries, as implemented in Gaussian. Since SODFT has a negligible effect on optimised structures, all geometry optimisations were conducted at the B3LYP level, while final electronic energies were obtained at the gB3LYP level.

Astatine was described using the aug-cc-pVTZ-pp basis set together with the Stuttgart relativistic effective core potential (ECP60MDF),^6^ which accounts for relativistic and spin–orbit effects. Lighter atoms were treated with the aug-cc-pVTZ-pp basis set.^7–9^ Due to the large atomic number of astatine, Grimme’s D3(BJ) dispersion correction was also applied.^10,11^

Solvent effects were introduced using the SMD implicit solvation model (water).^12^ The atomic cavity radius of At was set to 2.0485 Å for neutral/cationic species and 2.41 Å for anionic species.^13^ Strict convergence criteria were applied: force convergence of 1 × 10⁻⁴ hartree, energy convergence of 1 × 10⁻⁶ hartree, and displacement convergence of 1 × 10⁻⁵ hartree.

The resulting Gibbs free energies were used to construct the Eh–pH (Pourbaix) diagram and to evaluate the redox-dependent speciation of astatine (Fig. 4 and Table S2 ESI).

S2.2. Construction and interpretation of the Pourbaix diagram

The Pourbaix diagram of astatine species was constructed at 310 K using DFT-derived solvation free energies (G_solv), which were initially calculated in hartree and converted into kcal per mole to ensure thermodynamic consistency. The stability domains of astatine species were evaluated as a function of pH from 0 to 14 and electrode potential in the range of −1 to 2 V versus the standard hydrogen electrode. The following concentrations were adopted in the model: [At^−^] = 2.7 × 10^−6^ M, [other At species] = 1.0 × 10^−6^ M, [H_2_O_2_] = 5.0 × 10^−4^ M, [O_2_] = 2.36 × 10^−4^ M, with an oxygen partial pressure of 0.21 atm. The Nernst slope was corrected for temperature, giving a value of 0.0615 V per pH unit at 310 K.

The thermodynamic stability window of water is bounded by the hydrogen evolution line (E = 0 − 0.0615 × pH) and the oxygen evolution line (E = 1.174 − 0.0615 × pH, adjusted for dissolved oxygen). All astatine-related redox boundaries were derived from half-reactions involving two electrons unless otherwise stated. Within this window, three major stability regions can be identified. At high potentials above approximately E = 0.72 − 0.0615 × pH, astatine is stabilised predominantly as AtO^2−^ (At III), corresponding to oxidising conditions. Further oxidation to AtO^3−^ (At V) becomes accessible at even higher potentials. The conversion of AtO^−^ to AtO^2−^ is thermodynamically disfavoured, with a positive Gibbs free energy change of approximately +8.35 kcal mol^−1^, indicating that spontaneous oxidation is limited in the absence of strong oxidants.

At intermediate potentials between approximately E = 0.52 − 0.0615 × pH and E = 0.72 − 0.0615 × pH, the dominant species is AtO^−^ (At I), which represents a relatively stable intermediate oxidation state bridging the oxidised and reduced forms of astatine. The hydrolysis reaction At^+^ + H_2_O → AtO^−^ + 2 H^+^ is thermodynamically unfavourable near neutral pH, with a Gibbs free energy change of around +28.3 kcal mol^−1^, but becomes increasingly favourable in alkaline conditions (pH ＞10.4), explaining the enhanced stability of AtO^−^ at high pH.

At low potentials below approximately E = 0.52 − 0.0615 × pH, astatine is stabilised mainly as At^−^ (At −I), corresponding to strongly reducing conditions and showing behaviour analogous to that of iodide. The reduction of At^+^ to At^−^ is thermodynamically favourable, with a Gibbs free energy change of approximately −4.5 kcal mol^−1^. At pH values above about 8.5, the hydrogen evolution reaction begins to compete with the stability of At^−^. Because most equilibrium boundaries exhibit a similar slope of −0.0615 V per pH unit, the stability regions extend in parallel across the full pH range without significant intersections. In areas where two equilibria share a common species, such as AtO^2−^, the dominant form was determined by minimisation of the Gibbs free energy.

To enable direct comparison with astatine, the redox behaviour of iodide species was analysed using the same thermodynamic and computational framework applied to astatine. The resulting Gibbs free energies were used to generate the iodide redox-couple plots and Pourbaix predominance diagram presented in Fig. 4c,d of the main text.

The iodide model included the principal aqueous iodine species relevant to oxidation in biological and acidic environments, namely I⁻, IO⁻, IO₂⁻, IO₃⁻, and IO₄⁻. Standard redox half-reactions connecting these species were defined, and pH-dependent electrode potentials were derived from the corresponding reaction Gibbs free energies. All potentials were referenced to the standard hydrogen electrode (SHE). Water-stability boundaries, including H_2_O/H_2_, O_2_/H_2_O, and H_2_O_2_/H_2_O where appropriate, were included in the analysis.

The iodide redox analysis was performed over the same pH range and under the same thermodynamic conventions used for astatine, thereby allowing a matched theoretical comparison between the two systems.

S3. Supplementary Figures


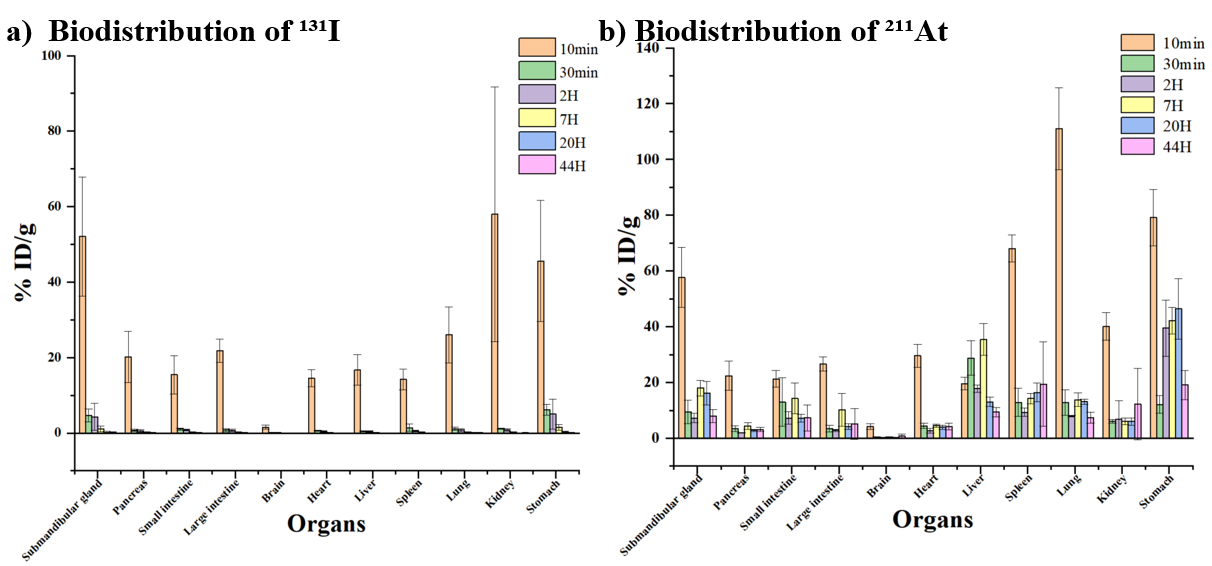


**Fig. S1** Comparative biodistribution and pharmacokinetics of Na²¹¹At and Na¹³¹I in BALB/c mice. (a) Na²¹¹At and (b) Na¹³¹I in key organs of female BALB/c mice (n=3) after intravenous administration. Tissues were harvested at 10 min, 30 min, 2 h, 7 h, 20 h, and 44 h post-injection. All radioactivity measurements were decay-corrected for the respective physical half-lives of ²¹¹At (7.214 h) and ¹³¹I (8.025 days) to accurately reflect pharmacokinetic profiles.​ Data represent mean ± SD. The distinct temporal profiles underscore the rapid clearance and minimal retention of ¹³¹I, in stark contrast to the prolonged and distinct accumulation of ²¹¹At in non-target organs such as the stomach, spleen, liver and lungs.


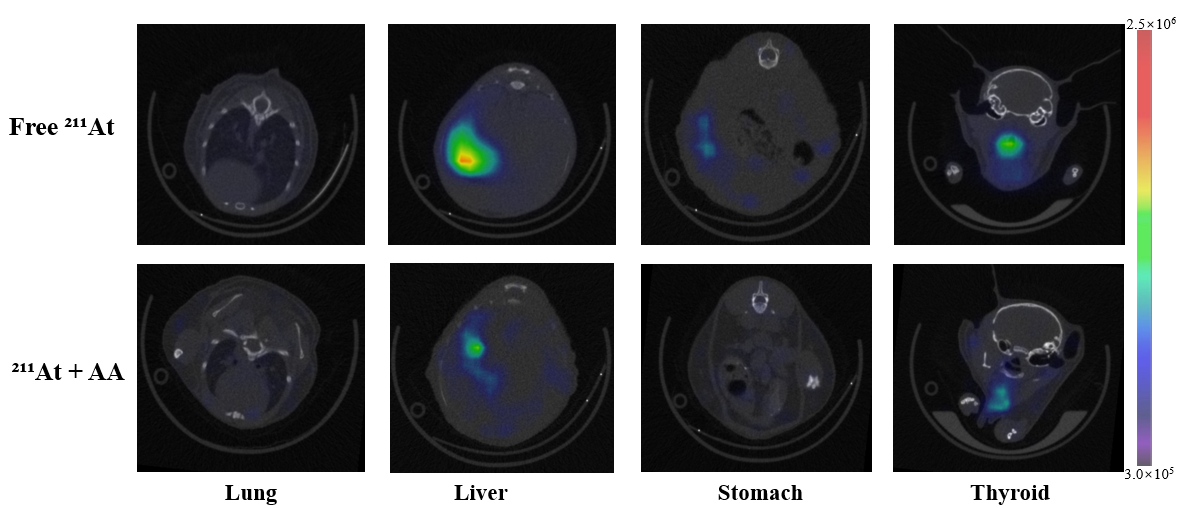


**Fig. S2** Representative transverse micro-SPECT/CT images of BALB/c mice (n = 3 per group) showing the distribution of ²¹¹At in the liver, lung, stomach, and thyroid at 2 h after intravenous injection. The upper row shows mice injected with free ^211^At, whereas the lower row shows mice co-injected with ^211^At and sodium ascorbate (^211^At + AA). Reduced signal intensity is observed in several transverse sections following co-administration of sodium ascorbate. Images are displayed using the same intensity scale (Bq/mL) within this figure, as indicated by the corresponding colour bar.


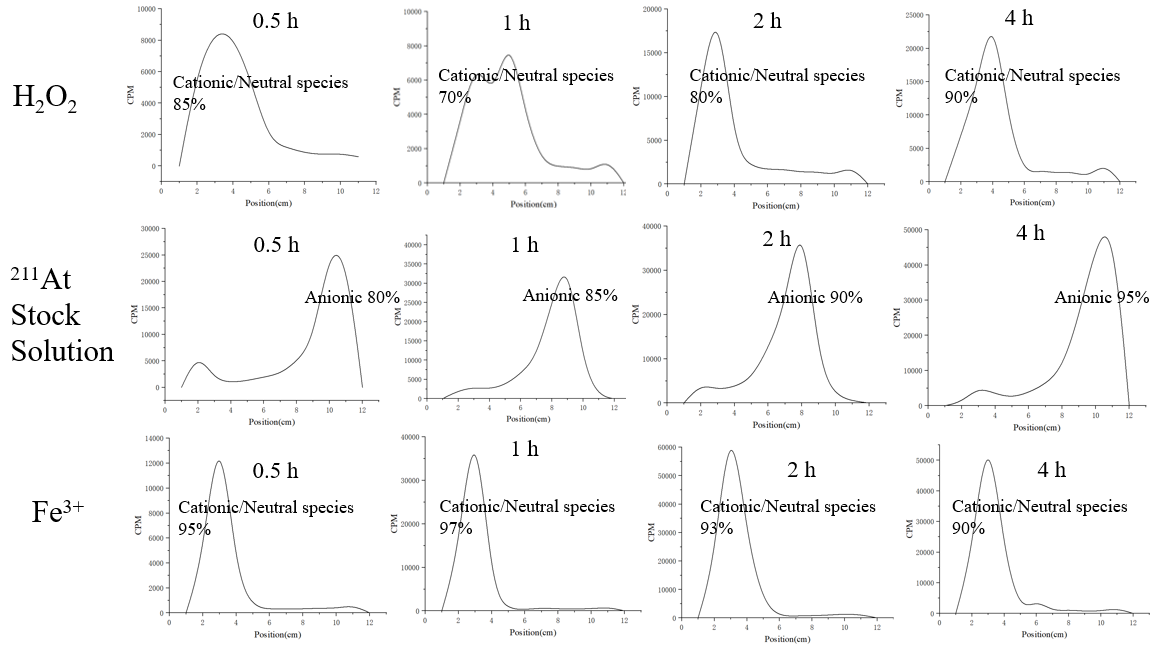


**Fig. S3** Time-dependent oxidation of ²¹¹At by H₂O₂ and Fe³⁺ analyzed by radio-thin layer chromatography (radio-TLC). Free ²¹¹At was incubated in FBS at 37 °C with H₂O₂ (500 μM) or Fe³⁺ (100 μM), and aliquots were analyzed by radio-TLC (iTLC-SG, 0.9% saline mobile phase) at 0.5, 1, 2, and 4 h. A ²¹¹At stock-solution control was analyzed in parallel. Oxidant-treated samples showed predominant low-mobility cationic/neutral species, whereas the stock solution remained mainly in the anionic fraction. Percent values denote the approximate proportion of the dominant fraction in each chromatogram.


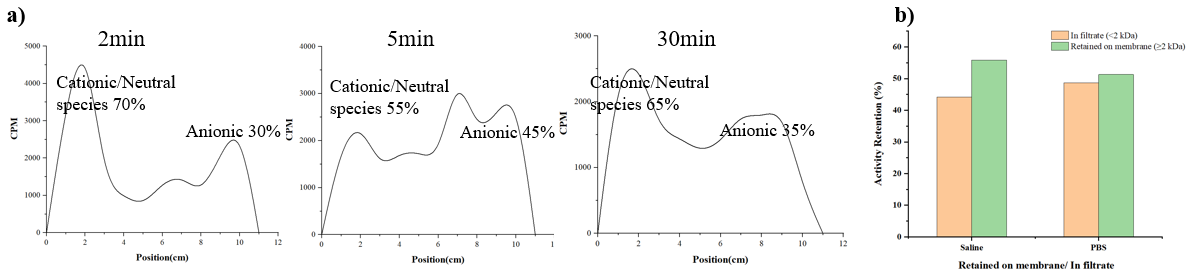


**Fig. S4** Colloid formation and ultrafiltration (2 kDa cutoff). (a) Radio-TLC profiles of Na²¹¹At in saline at 2, 5, and 30 min, showing the rapid decrease of the free At⁻ fraction and the concurrent formation of less mobile species. (b) Ultrafiltration of Na²¹¹At after 15 h incubation in saline or PBS (2 kDa cutoff). A substantial proportion of activity was retained on the membrane, indicating the presence of colloidal or aggregated hydrolyzed ²¹¹At species.


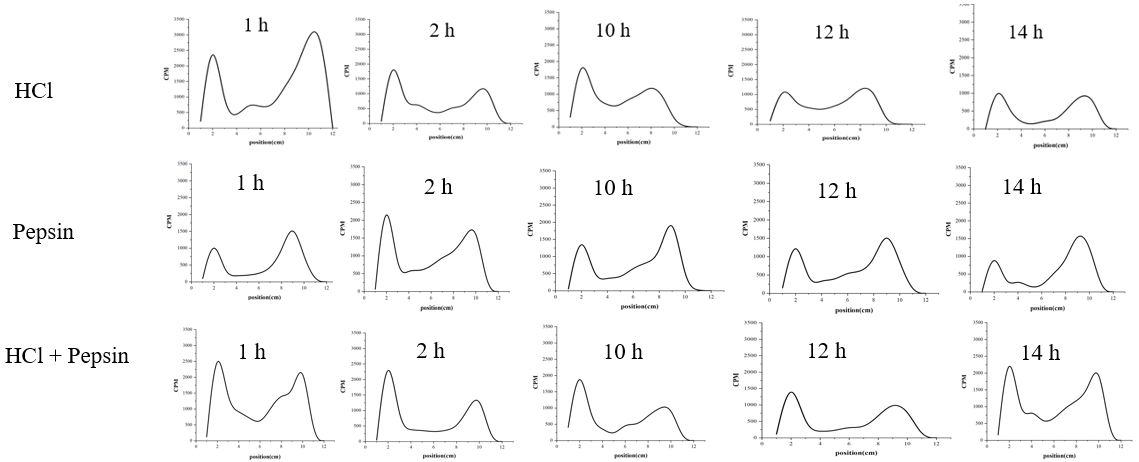


**Fig. S5** ²¹¹At oxidation kinetics under simulated gastric conditions. TLC analysis shows time-dependent conversion of ²¹¹At from anionic (At⁻) to cationic species in HCl and HCl+Pepsin environments, confirming acid-driven oxidation. Pepsin alone induces minimal change.


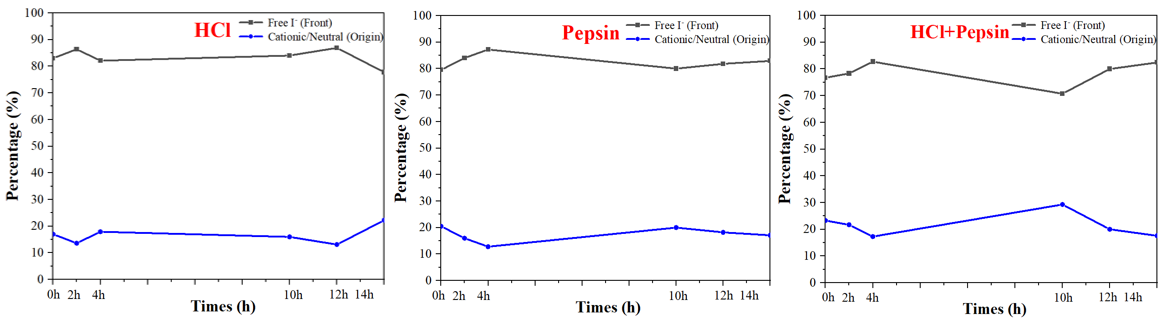


**Fig. S6** ¹³¹I speciation under simulated gastric conditions. Radio-TLC profiles of Na¹³¹I incubated in HCl, pepsin, or HCl+pepsin at 37 °C. Aliquots collected at 0, 1, 2, 10, 12, and 14 h show no appreciable formation of non-anionic iodine species under these conditions, in contrast to the time-dependent oxidation observed for ²¹¹At (Fig. S5).


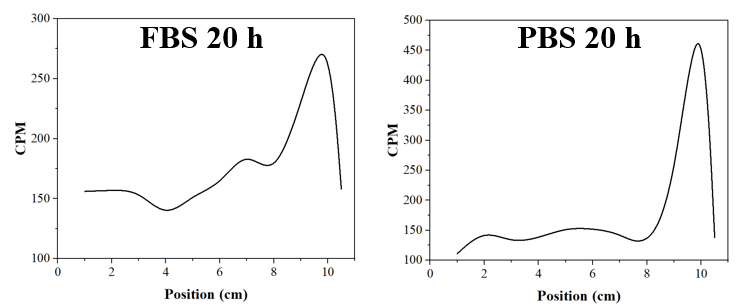


**Fig. S7** Stability of ¹³¹I in FBS and PBS after 20 h incubation at 37 °C. Radio-TLC profiles of Na¹³¹I incubated in FBS (left) and PBS (right) for 20 h at 37 °C. In contrast to ²¹¹At, no detectable formation of non-anionic or colloidal iodine species was observed. The activity remained predominantly at the solvent front, consistent with stable iodide (I⁻) under physiological conditions.

S4. Supplementary Tables

| Fraction | Activity (CPM) | Percentage of Total (%) |
| --- | --- | --- |
| QMA cartridge retained (anionic) | 151,496 ± 9,210 | 54.75 ± 3.21 |
| C18 cartridge retained (hydrophobic) | 116,392 ± 7,085 | 34.77 ± 2.58 |
| Final filtrate (hydrophilic) | 66,844 ± 4,965 | 19.97 ± 1.85 |

**Table S1** Sequential cartridge analysis of ²¹¹At species. The percentage of total activity retained on the QMA cartridge (anionic species), C18 cartridge (hydrophobic species), and remaining in the final filtrate (hydrophilic species) is shown for ²¹¹At after incubation. The distribution reflects the coexistence of anionic, hydrophobic, and hydrophilic astatine species.

| Species | G_solv_ (hartree) |
| --- | --- |
| At^-^ | −262.8634 |
| At^+^ | −262.5009 |
| AtO⁺ | −337.6640 |
| AtO⁻ | −338.0302 |
| AtO₂⁻ | −413.2251 |
| AtO₃⁻ | −488.4349 |
| AtO₄⁻ | −563.5475 |
| AtO(OH) | −413.6941 |
| AtO(OH)₂⁻ | −489.6903 |

**Table S2** Calculated solvation free energies (Gₛₒₗᵥ) of astatine species at the B3LYP/SMD level (310 K). Solvation free energies (Gₛₒₗᵥ) were computed using B3LYP with the Stuttgart ECP60MDF effective core potential and aug-cc-pVTZ-pp basis sets for At, and aug-cc-pVTZ-pp for lighter elements, within the SMD solvation model (water). Free energies are reported without thermal corrections unless otherwise specified.

| Species | G_solv_ (hartree) |
| --- | --- |
| IO^-^ | -371.18 |
| IO_2_^-^ | −446.36 |
| IO_3_^-^ | −521.57 |
| IO_4_^-^ | −596.70 |
| H_2_ | −1.17 |
| O_2_ | −150.25 |
| H_2_O | −76.43 |
| H_2_O_2_ | −151.53 |
| OH^-^ | −75.74 |
| H^+^ | -0.43 |

**Table S3** Calculated solvation free energies (G_solv_) of iodide-related species and reference molecules at the matched theoretical level (310 K). Solvation free energies (G_solv_) of aqueous iodine species (I-containing oxyanions) together with reference molecules and ions were used to derive the redox half-reactions and construct the iodide Pourbaix diagram shown in Fig. 4c,d. Calculations were performed under the same thermodynamic framework and solvation model used for the astatine analysis to allow direct redox comparison. Free energies are reported in hartree without thermal corrections unless otherwise specified.

| Group | Mouse | ALT (U/L) | AST (U/L) | ALP (U/L) | CREA (μmol/L) | UREA (mmol/L) |
| --- | --- | --- | --- | --- | --- | --- |
| Control​ | #1 | 35.4 | 177.2 | 200.6 | 12.8 | 8.8 |
|  | #2 | 42.2 | 127.9 | 201.8 | 17.8 | 7.7 |
|  | #3 | 38.8 | 201.6 | 232.5 | 14.6 | 6.7 |
|  | Mean ± SD​ | 38.8 ± 3.4​ | 168.9 ± 37.5​ | 211.6 ± 18.1​ | 15.1 ± 2.5​ | 7.8 ± 1.1​ |
| Acute Injury | #1 | 36.2 | 112.8 | 193.1 | 15.2 | 5.9 |
|  | #2 | 37.6 | 113 | 175.3 | 13.8 | 5.7 |
|  | #3 | 60.9 | 160.8 | 198.5 | 15 | 4.9 |
|  | Mean ± SD​ | 44.9 ± 13.9​ | 128.9 ± 27.7​ | 189.0 ± 12.1​ | 14.7 ± 0.8​ | 5.5 ± 0.6 |
| Long-term Injury | #1 | 44.5 | 126.4 | 163.3 | 13.4 | 5.6 |
|  | #2 | 58.3 | 119.6 | 125.3 | 9.4 | 5.5 |
|  | #3 | 45 | 151.3 | 206.7 | 11 | 5.7 |
|  | Mean ± SD​ | 49.3 ± 7.8​ | 132.4 ± 16.7​ | 165.1 ± 40.7​ | 11.3 ± 2.0​ | 5.6 ± 0.1 |

**Table S4** Serum biochemical parameters following ²¹¹At administration. Serum levels of ALT, AST, ALP, creatinine (CREA), and urea (UREA) in control, acute-injury (day 3), and long-term-injury (day 15) mice following administration of ²¹¹At. Data are presented as individual values and mean ± SD (n = 3 per group).

**Reference**

1 K. Yaginuma, K. Takahashi, S. Hoshi, T. Joho, S. Shimoyama, N. Hasegawa, K. Hasegawa, S. Zhao, N. Ukon, S. Makabe, S. Meguro, A. Onagi, K. Matsuoka, S. Ogawa, M. Uemura, T. Yamashita, H. Suzuki, T. Uehara and Y. Kojima, Eur. J. Nucl. Med. Mol. Imaging, 2025, 52, 469–481.

2 T. Saue, ChemPhysChem, 2011, 12, 3077–3094.

3 J. Champion, A. Sabatié-Gogova, F. Bassal, T. Ayed, C. Alliot, N. Galland and G. Montavon, J. Phys. Chem. A, 2013, 117, 1983–1990.

4 J. Champion, C. Alliot, E. Renault, B. M. Mokili, M. Chérel, N. Galland and G. Montavon, J. Phys. Chem. A, 2010, 114, 576–582.

5 J. Champion, M. Seydou, A. Sabatié-Gogova, E. Renault, G. Montavon and N. Galland, Phys. Chem. Chem. Phys., 2011, 13, 14984.

6 K. A. Peterson, D. Figgen, E. Goll, H. Stoll and M. Dolg, J. Chem. Phys., 2003, 119, 11113–11123.

7 T. H. Dunning, J. Chem. Phys., 1989, 90, 1007–1023.

8 R. A. Kendall, T. H. Dunning and R. J. Harrison, J. Chem. Phys., 1992, 96, 6796–6806.

9 D. E. Woon and T. H. Dunning, J. Chem. Phys., 1993, 98, 1358–1371.

10 S. Grimme, J. Antony, S. Ehrlich and H. Krieg, J. Chem. Phys., 2010, 132, 154104.

11 S. Grimme, S. Ehrlich and L. Goerigk, *J. Comput. Chem.*, 2011, **32**, 1456–1465.

12 A. V. Marenich, C. J. Cramer and D. G. Truhlar, *J. Phys. Chem. B*, 2009, **113**, 6378–6396.

13 J. Champion, A. Sabatié-Gogova, F. Bassal, T. Ayed, C. Alliot, N. Galland and G. Montavon, *J. Phys. Chem. A*, 2013, **117**, 1983–1990.
