# Supplementary material for: Redox-driven speciation and colloid formation contribute the in vivo chemistry and organ deposition of Astatine-211
Source: EJNMMI Radiopharm Chem. 2026 Mar 31;11:34. doi: 10.1186/s41181-026-00441-3 (PMC13172141; doi:10.1186/s41181-026-00441-3)
Supplement: Supplementary file 2 — Supplementary Material 2. [file 41181_2026_441_MOESM2_ESM.docx]

Manuscript Title: Redox-Driven Speciation and Colloid Formation Contribute the *In Vivo* Chemistry and Organ Deposition of Astatine-211

Authors: Xiaoxu Dong, Jiaojiao Li, Mumei Chen, Kaiqi Zhang, Xiaozheng Zhang, Fengling Shan, Shuai Xue, Xiao Li, Lan Zhang, Huawei Cai, Jiajun Liu, and Qingnuan Li

Manuscript Number: ERPC-D-25-00168

Dear Dr. Philip H. Elsinga and Reviewers:

We would like to sincerely thank you for your time and effort in evaluating our manuscript and for the constructive comments and helpful suggestions. We are grateful for the reviewers’ thoughtful feedback, which has significantly helped us improve the clarity, rigor, and overall quality of the manuscript.

We have carefully considered all comments and revised the manuscript accordingly. For clarity, all revisions in the manuscript have been highlighted in red text. Below we provide a detailed, point-by-point response to each comment.

We hope that the revisions and our responses adequately address the reviewers’ and editor’s concerns. We look forward to your favorable consideration.

Yours sincerely,

Xiaoxu Dong

Shanghai Institute of Applied Physics, Chinese Academy of Sciences, Shanghai 201800, China

E-mail: dongxiaoxu@sinap.ac.cn

*************************************************************************

## **Point by Point Response to the Reviewers’ Comments:**

(Reviewer comments are reproduced in blue italics, followed by our responses in black text. “Fig. x” and “Fig. Sx” refer to figures in the revised main text and ESI, respectively.)

## **Response to Reviewer # 1**

1. *The overall use of the adjective 'aberrant' throughout the article for the biodistribution of astatine species should be reconsidered. The fact that the biodistribution is different from the iodine species does not necessarily mean it is aberrant or abnormal, it is not aberrant but divergent or characteristic. Astatine is a different nuclide with different properties (e.g. semimetal), and thus it is unsurprising to observe differences when compared to iodine. The language should be adequate to this observation.*

**Response:**

We fully agree with the reviewer. Throughout the manuscript, the term “aberrant” has been replaced with more neutral descriptors such as “distinct,” “divergent,” “characteristic,” or “non-target” to reflect physicochemical differences rather than abnormal behavior. This change has been implemented in the revised version.

2. *It is not clear if the authors refer to astatine-211 as the nuclide in its free form (astatine) at the first mention of the element as a therapeutic candidate (line 44). It seems like it but directly after and without any transition there is mention of "^211^At-labeled compounds". Please make the differentiation between free astatine for thyroid-targeted treatment, analogous to radio-iodine, and radiolabelled compounds clearer.*

**Response:**

We have carefully revised the opening Background section to explicitly differentiate between free astatine-211 administered as astatide for NIS-mediated uptake, analogous to radioiodine therapy, and ²¹¹At incorporated into targeted radiopharmaceutical constructs. This distinction is now clearly introduced at the first mention of astatine as a therapeutic candidate and consistently maintained throughout the revised manuscript, in order to avoid conceptual ambiguity and to clarify the distinct biological contexts of free astatine and ²¹¹At-labelled radiopharmaceuticals.

3. *Not only PSAt-3-Ga by El Fakiri et al. has been developed for PSMA, the reader would benefit from the inclusion and/or reference of more candidates such as the ones developed by Watabe et al. and Mease et al.*

**Response:**

We thank the reviewer for highlighting the need to broaden the background discussion of PSMA-targeted ²¹¹At radiopharmaceuticals. In the revised manuscript, we have expanded this section to include additional representative studies beyond PSAt-3-Ga. Specifically, we now cite the recent first-in-human SPECT/CT imaging of [²¹¹At]PSMA-5 reported by Watabe et al., demonstrating clinical translational feasibility, as well as the improved PSMA-targeted ²¹¹At therapeutic agent developed by Mease et al., showing enhanced *in vivo* stability and therapeutic performance. These additions provide a more comprehensive and up-to-date overview of current advances in astatine-based PSMA radiopharmaceutical development and better contextualize the motivation for the present mechanistic study.

Page: 5-6 line: 85-100

Reference:

Watabe T, Hatano K, Naka S, Sasaki H, Kamiya T, Shirakami Y, et al. First-in-human SPECT/CT imaging of [^211^At]PSMA-5: targeted alpha therapy in a patient with refractory prostate cancer. Eur J Nucl Med Mol Imaging. 2025 June;52(7):2253–5.

Mease RC, Kang CM, Kumar V, Banerjee SR, Minn I, Brummet M, et al. An Improved ^211^At-Labeled Agent for PSMA-Targeted α-Therapy. J Nucl Med. 2022 Feb;63(2):259–67.

Naka S, Watabe T, Shirakami Y, Ooe K, Kurimoto K, Sakai T, et al. Scale-up production of [^211^At]PSMA-5 using automated synthesizer for Investigator-Initiated clinical trial. EJNMMI radiopharm. chem. 2025 Dec 27;11(1):6.

4. *Authors claim that "models extrapolated from iodide fail to predict its biodistribution" -Please provide references backing this claim, cite work from Forssell-Aronsson et al. (Cancer Biotherapy and Radiopharmaceuticals, 2006 and 2013)*

**Response:**

We have now added citations to Forssell-Aronsson et al. (Cancer Biotherapy and Radiopharmaceuticals, 2006 and 2013), to better substantiate that iodide-based pharmacokinetic expectations do not adequately account for the observed biodistribution of free astatine species.

Page: 6 line: 110

Reference:

Oddstig J, Bernhardt P, Jacobsson L, Forssell-Aronsson E. A Novel Photon Radiation Detector System for *In Vitro* Biokinetic Measurements. Cancer Biotherapy and Radiopharmaceuticals. 2005 Dec;20(6):629–38.

Spetz J, Rudqvist N, Forssell-Aronsson E. Biodistribution and Dosimetry of Free ^211^At,^125^ I^−^ and^131^ I^−^ in Rats. Cancer Biotherapy and Radiopharmaceuticals. 2013 Nov;28(9):657–64.

5. *SPECT imaging: it is not clear if the mice received the intravenous administration under anaesthesia and if the mice were left under anaesthesia for 2 hours until the scan time, or if they received the injection under anaesthesia and then were free to roam until scan time, or if they received the injection without prior anaesthesia and were then anaesthetised for the imaging procedure. Please clarify.*

**Response:**

We agree with the reviewer that the anaesthesia/injection timeline should be explicit. We have therefore revised Section 2.4 (Micro-SPECT/CT imaging) to clearly state the exact workflow: radiotracers were administered by tail-vein injection in awake mice without prior anaesthesia; animals were then returned to their cages and allowed to move freely until the scheduled imaging time point (2 h post-injection); and anaesthesia was induced only immediately before SPECT/CT acquisition and maintained exclusively for the duration of image acquisition.

Page: 10-11 line: 195-206

6. *Speciation analysis: it is not clear how the samples were processed after incubation. Please specify the procedure followed not only in conditioning the SPE cartridges but also when applying and processing the samples.*

**Response:**

We thank the reviewer for pointing out the need for a clearer description of the SPE-based speciation workflow. The Methods section has now been substantially expanded to explicitly detail each step of cartridge conditioning, sample loading, washing, fraction collection, and radioactivity quantification. Specifically, we now describe this in the revised version as follow.

Page: 12-13 line: 238-262

(i) the full pre-conditioning procedures for both QMA anion-exchange and C18 cartridges, including solvent compositions and volumes;

(ii) the sequential loading of incubated samples onto QMA cartridges, followed by defined water rinsing steps to separate anionic from non-anionic species;

(iii) subsequent processing of the QMA flow-through on C18 cartridges to distinguish hydrophilic and hydrophobic fractions; and

(iv) quantitative recovery and decay-corrected γ-counting of all fractions.

These additions clearly delineate how anionic (QMA-retained), neutral/cationic (QMA flow-through), and hydrophobic/protein-associated (C18-retained) astatine species were separated and analyzed. These additions improve methodological transparency and reproducibility.

7. *TLC: which solid support was used to run the TLC analyses? Normal phase? Reverse phase? iTLC? Please specify.*

**Response:**

We thank the reviewer for highlighting the need to clarify the TLC methodology. In the revised manuscript, all TLC experiments are now explicitly described as radio-TLC performed on Agilent iTLC-SG strips (glass microfiber chromatography paper impregnated with silica gel; Agilent Technologies) using 0.9% physiological saline (0.9% NaCl) as the mobile phase. This TLC system (silica gel stationary phase with aqueous saline eluent) is applied consistently throughout the study, including radiochemical purity assessment and all speciation analyses. We have standardised the terminology to “radio-TLC (iTLC-SG; 0.9% saline mobile phase)” at each occurrence in the Methods to ensure methodological transparency and consistency.

Page: 12 line: 229-232

8. *Imaging - Figure 1: the image shows biodistribution of iodine and astatine species while the caption only mentions astatine, please amend accordingly. Provide colour scale indicating PET SUV values.*

**Response:**

We thank the reviewer for this important clarification. The caption of Figure 1 has been thoroughly revised to accurately reflect all experimental conditions shown in the images. Specifically, we now explicitly indicate the administration of free ¹³¹I, ¹³¹I + ascorbic acid (¹³¹I + AA), free ²¹¹At, and ²¹¹At + ascorbic acid (²¹¹At + AA), together with the injected activities, imaging time point (2 h post-injection), and the number of animals per group (n = 3).

In addition, intensity colour bars have now been added to Figures 1 and S2. Since the imaging data were acquired by SPECT/CT (rather than PET), the colour scales are presented in Bq/mL, as indicated in the revised figure legends. Because ¹³¹I and ²¹¹At were scanned on different SPECT systems, signal intensities are not intended for direct quantitative comparison between radionuclides.

**Fig. 1** *In vivo* distribution and proposed mechanisms of ²¹¹At organ accumulation. (a) Representative whole-body micro-SPECT/CT images of BALB/c mice (n = 3 per group) acquired at 2 h after intravenous administration of free ¹³¹I (100 µCi), ¹³¹I + ascorbic acid (¹³¹I + AA), free ²¹¹At (50 µCi), and ²¹¹At + ascorbic acid (²¹¹At + AA), arranged from left to right. Images are displayed using radionuclide-specific intensity scales (Bq/mL), as indicated by the corresponding colour bars. Because ¹³¹I and ²¹¹At were acquired on different SPECT systems, signal intensities are not intended for direct quantitative comparison between radionuclides. (b) Schematic illustrating the proposed organ-specific mechanisms of off-target ²¹¹At deposition, including reactive oxygen species (ROS)-driven oxidation to cationic species (e.g., AtO⁺) in the lungs; hydrolysis and colloidal formation in the liver and spleen; and acidic hydrolysis to At⁺ in the stomach.

9. *Imaging - Figure S2: image and caption contradict, please amend accordingly. Provide colour scale indicating PET SUV values.*

**Response:**

We thank the reviewer for identifying this inconsistency. In the revised ESI, the caption of Fig. S2 has been corrected to accurately match the image order, with the upper row corresponding to free ²¹¹At and the lower row to the ²¹¹At + ascorbic acid (AA) co-administration group.

In addition, intensity colour scale bars have now been added to all SPECT/CT images in Figure S2 (ESI; page 6, lines 171-176) to facilitate visual comparison of tracer distribution and relative signal intensity. As these images were acquired by SPECT/CT rather than PET, the colour scales are presented in Bq/mL, as indicated in the revised figure legend. These revisions improve clarity and ensure consistency between the images and their descriptions.

**Fig. S2** Representative transverse micro-SPECT/CT images of BALB/c mice (n = 3 per group) showing the distribution of ²¹¹At in the liver, lung, stomach, and thyroid at 2 h after intravenous injection. The upper row shows mice injected with free ²¹¹At, whereas the lower row shows mice co-injected with ²¹¹At and sodium ascorbate (²¹¹At + AA). Reduced signal intensity is observed in several transverse sections following co-administration of sodium ascorbate. Images are displayed using the same intensity scale (Bq/mL) within this figure, as indicated by the corresponding colour bar.

10. *Critical observation on mechanistic hypotheses: the mechanistic argument is built on experiments performed only on ^211^At. This is a fundamental weakness of the experimental design and hypothesis confirmation strategy. It is inaccurate to claim that the observed biodistribution differences are due to unique astatine chemistry without comparing the same experiments carried out with iodine-131.*

**Response:**

We thank the reviewer for this important point and agree that mechanistic interpretation should not rely solely on ²¹¹At data when the central claim concerns chemical divergence from iodide. In response, we have strengthened the revised manuscript by incorporating matched iodide controls and an explicit computational comparison with iodide, and we have integrated these additions into the Results/Discussion/Conclusion.

Specifically, we now include: (i) an additional ¹³¹I + ascorbic acid (AA) imaging control in Fig. 1a; (ii) a matched ¹³¹I simulated gastric incubation control demonstrating iodide stability across HCl, pepsin, and HCl/pepsin conditions (Fig. S6, ESI; page 8, lines 199-203); (iii) a matched ¹³¹I incubation in FBS and PBS showing no detectable formation of less mobile or non-anionic iodine species by radio-TLC (Fig. S7, ESI; page 8, lines 204-208); and (iv) an explicit computational redox comparison of astatine versus iodide incorporated into the revised main text (Fig. 4; page 25, lines 459–502). Together, these additions substantiate that the observed divergence between ²¹¹At and ¹³¹I is consistent with chemically distinct redox behavior rather than a generic halide effect.

**Fig. 4** Theoretical redox analysis of astatine and iodide under physiological conditions. (a) Calculated redox-couple potentials of ²¹¹At species as a function of pH and electrode potential (E, vs. the standard hydrogen electrode, SHE), with physiological gastric and lung pH ranges indicated. (b) Calculated Pourbaix predominance diagram of ²¹¹At, showing the stability domains of At⁻, At⁺, AtOOH, AtO⁻, and AtO₂⁻ under aqueous conditions. (c) Calculated redox-couple potentials of iodide species under the same theoretical framework, with physiological gastric and lung pH ranges indicated. (d) Calculated Pourbaix predominance diagram of iodide, showing the stability domains of I⁻, IO⁻, IO₃⁻, IO₄⁻, and IO₂⁻ under aqueous conditions. Together, these calculations highlight the markedly greater redox sensitivity of astatine relative to iodide and provide a theoretical basis for their distinct *in vivo* chemical behaviors.

11. *In all cases authors should perform the same experiments: oxidation by H_2_O_2_/Fe^3+^, incubation and filtration (colloid formation) and simulated gastric conditions with iodine-131. This is a necessary control to further support or refute the hypotheses of the authors. Without such controls it is difficult to affirm that the differential biodistribution of astatine-211 when compared to iodine-131 is due to the formation of cationic species and/or colloids. It is especially important in the filtration assay in which colloid/aggregate formation is claimed.*

**Response:**

We thank the reviewer and agree that fully matched iodide controls across all mechanistic assays would provide the strongest validation. In the present revision, we have addressed the most direct component of this request by adding the matched simulated gastric-condition iodide experiments (Fig. S6, ESI) and by adding matched iodide incubation controls in FBS and PBS (Fig. S7, ESI), which together demonstrate that iodide remains predominantly anionic and does not form detectable non-anionic/less mobile species under conditions where ²¹¹At shows clear transformation.

We acknowledge that a fully matched dataset for (i) side-by-side H₂O₂/Fe³⁺ oxidation series for ¹³¹I using the same TLC/SPE workflow, and (ii) side-by-side ultrafiltration/filtration assays for ¹³¹I under multiple media and oxidant conditions has not yet been completed in this revision. Accordingly, we have moderated the corresponding mechanistic wording in the revised manuscript and now interpret the retained fraction conservatively as evidence for colloid-like/macromolecular astatine species, rather than attributing it exclusively to inorganic colloids.

12. *Moreover, the filtration experiments would benefit from performing an incubation in protein-rich medium such as mouse serum or FBS, rather than just PBS.*

**Response:**

We thank the reviewer for this helpful suggestion and agree that protein-rich incubation conditions would further strengthen interpretation of the filtration-based results. In the revised manuscript, we have therefore more explicitly incorporated the role of protein-rich environments into the mechanistic discussion. Complementary FBS incubation experiments showed that ²¹¹At undergoes transformation in a protein-rich medium, consistent with the formation of non-anionic and protein-associated species, whereas matched Na¹³¹I controls incubated in FBS and PBS remained stable by radio-TLC and did not show evidence of less mobile or non-anionic species (Fig. S7, ESI).

At the same time, we considered that direct ultrafiltration in FBS would introduce multiple overlapping factors that would complicate mechanistic interpretation. In addition to oxidation-related transformation, ²¹¹At may undergo additional interactions with serum proteins, including binding at disulfide-containing protein sites, hydrophobic domains, and coordination/binding sites, such that membrane retention could arise from protein association or complex formation rather than colloid formation alone. For this reason, PBS was used as a simplified medium for the ultrafiltration assay, allowing clearer evaluation of colloid-like/macromolecular fraction formation under controlled conditions. Accordingly, we have kept the wording in the revised manuscript cautious and now interpret the retained fraction as evidence for colloid-like/macromolecular astatine species, rather than attributing it exclusively to inorganic colloid formation.

13. *Authors perform in vivo experiments with co-administration of ascorbic acid but did not perform in vitro assays in presence of ascorbic acid, performing the in vitro assays in presence of ascorbic acid would be beneficial.*

**Response:**

We thank the reviewer for this constructive suggestion and agree that in vitro assays performed in the presence of ascorbic acid (AA) would further strengthen the mechanistic interpretation. In the present revision, we did not add a full *in vitro* AA-treated speciation series across all analytical platforms. However, to address this point, we have now cited the work of Watabe et al. (J Nucl Med, 2019) in the revised manuscript (page 31, line 618), in which the antioxidant effect of AA on free ²¹¹At was experimentally demonstrated *in vitro*.

In the context of the present study, our newly added *in vivo* AA control imaging supports a directionally consistent interpretation: co-administration of AA visibly altered the distribution of ²¹¹At, whereas the corresponding ¹³¹I + AA control did not show an obvious redistribution. Together with the literature evidence from Watabe et al., these findings support the view that AA can suppress redox-sensitive transformation of astatine.

At the same time, we agree that a fully matched *in vitro* AA-treated assay set would provide an additional level of mechanistic confirmation. Accordingly, we have kept the relevant discussion in the revised manuscript appropriately cautious and have not overextended the mechanistic claim beyond the currently available experimental and literature-supported evidence.

14. *Additional experimental work required:
    o Side-by-side in vitro speciation (TLC, SPE) of astatine and iodine after incubation with oxidants.
    o Side-by-side in vitro ultrafiltration of astatine-211 and iodine-131 after incubation in different mediums, including protein rich ones, and with/without oxidants.
    o In vitro speciation of astatine-211 and iodine-131 in gastric simulated fluid
    o In vitro speciation of both radionuclides through all described techniques with presence of ascorbic acid (alike to the in vivo work).*

**Response:**

We thank the reviewer for outlining this comprehensive set of suggested experiments and agree that a complete side-by-side matrix would further strengthen mechanistic validation. In this revision, we have focused on adding the most hypothesis-relevant iodide controls feasible within the revision timeframe, as detailed above (Comments 10–13), including matched gastric and FBS/PBS controls, an additional ¹³¹I + AA imaging control, and an explicit computational comparison with iodide. These additions directly address the reviewer’s central concern that key mechanistic conclusions should be evaluated against iodide under matched conditions.

We acknowledge that not all elements of the requested side-by-side matrix (e.g., full oxidant-series TLC/SPE for iodide, matched ultrafiltration under oxidants/media, and comprehensive in vitro AA-treated speciation across all platforms) are yet included in the present revision. We have therefore kept the revised interpretation appropriately cautious and aligned the Discussion/Conclusion strictly with the evidence currently available.

15. *Discussion and conclusion:
    - The discussion currently interprets the data in absence of the critical controls mentioned in the previous section, it needs to be adapted accordingly after performing the experiments.
    - Likewise, the conclusion of the manuscript needs to be amended and adapted after including the additional experiments.*

**Response:**

We thank the reviewer for this important comment and agree that both the Discussion and the Conclusion should be revised in light of the newly added control experiments. As described above, we have now incorporated additional iodide controls in simulated gastric media, FBS/PBS incubation conditions, AA-treated imaging, and theoretical comparison, and we have revised the Discussion and Conclusion accordingly to reflect this expanded evidence base.

In the revised Discussion, we now explicitly contrast astatine and iodide under matched conditions. Specifically, we note that: (i) under simulated gastric conditions, iodide remained predominantly in the anionic form without detectable oxidation, in contrast to the clear acid-promoted transformation observed for ²¹¹At (Fig. S6, ESI); (ii) under matched FBS/PBS incubation conditions, iodide remained stable by radio-TLC without evidence of less mobile or non-anionic species, whereas ²¹¹At underwent clear transformation in a protein-rich environment (Fig. S7, ESI); and (iii) the corresponding ¹³¹I + AA imaging control did not show an obvious redistribution relative to free ¹³¹I, supporting that the attenuation observed for ²¹¹At is more closely related to suppression of redox-sensitive astatine transformation than to a nonspecific effect of AA.

The Conclusion has also been amended accordingly and now explicitly reflects the matched iodide control data and the added theoretical comparison. Rather than framing the mechanistic interpretation on the basis of ²¹¹At alone, the revised conclusion now states that the *in vivo* chemistry of free astatine-211 is fundamentally distinct from that of iodide and that this distinction is supported by combined experimental, computational, and matched iodine-131 control evidence.

16. *The most critical deficiency is the lack of iodine-131 control experiments across all key in vitro mechanistic assays. Only after inclusion of these experiments and the clarifications in methodology, corrections to figures, and other minor comments addressed in this review; the manuscript can be considered for publication.*

**Response:**

We thank the reviewer for this clear summary and agree that iodide controls were essential to strengthen the mechanistic basis of the manuscript. In response, we have revised the manuscript extensively and added the principal iodide control experiments and methodological clarifications needed to strengthen the mechanistic comparison.

In brief, the revision includes: (i) matched iodide control experiments under simulated gastric and protein-rich incubation conditions (Figs. S6–S7, ESI), (ii) an explicit computational comparison with iodide incorporated into the revised main text (Fig. 4), and (iii) improved methodological and presentation clarity, including expanded imaging/TLC/SPE descriptions and corrected figure legends with intensity colour bars.

We acknowledge that a fully symmetrical iodide dataset across every mechanistic assay is not yet complete (particularly matched oxidant-series TLC/SPE and ultrafiltration controls). Accordingly, we have moderated the corresponding mechanistic wording throughout the revised manuscript and now present the conclusions in a more evidence-bounded manner. We believe these revisions substantially address the reviewer’s major concern and provide a stronger and more balanced basis for evaluation.

## **Response to Reviewer # 2**

We sincerely thank the reviewer for the positive evaluation of our work and for the constructive suggestions that helped improve the clarity and completeness of the manuscript. We have carefully revised the manuscript in accordance with all comments. Detailed responses are provided below.

1. *Some references in the Introduction appear to be outdated and could be updated to reflect more recent advances in the field.*

**Response:**

We thank the reviewer for this helpful suggestion. The Introduction has been revised to incorporate recent advances in astatine-211 radiopharmaceutical development and early clinical translation. In particular, we have added recent first-in-human imaging of [²¹¹At]PSMA-5 (Watabe et al., 2025), as well as improved PSMA-targeted ²¹¹At agents demonstrating enhanced *in vivo* stability and therapeutic performance (Mease et al., 2022). These additions provide a more up-to-date overview of the field and better contextualize the chemical challenges addressed in the present study.

Reference:

Watabe T, Hatano K, Naka S, Sasaki H, Kamiya T, Shirakami Y, et al. First-in-human SPECT/CT imaging of [^211^At]PSMA-5: targeted alpha therapy in a patient with refractory prostate cancer. Eur J Nucl Med Mol Imaging. 2025 June;52(7):2253–5.

Mease RC, Kang CM, Kumar V, Banerjee SR, Minn I, Brummet M, et al. An Improved ^211^At-Labeled Agent for PSMA-Targeted α-Therapy. J Nucl Med. 2022 Feb;63(2):259–67.

2. *Please include the half-lives of ²¹¹At and ¹³¹I for reference, as this would benefit readers less familiar with these radionuclides.*

**Response:**

We thank the reviewer for this valuable suggestion. The physical half-lives of both radionuclides have now been explicitly stated at their first mention in the Background section (²¹¹At, t₁/₂ = 7.2 h; ¹³¹I, t₁/₂ = 8.0 d), thereby providing clearer radiophysical context for readers.

Page: 4 lines: 66-70

3. *SPECT intensity scale bars should be included in Figure 1A.*

**Response:**

We thank the reviewer for this helpful suggestion. Intensity scale bars have now been added to all micro-SPECT/CT images in Figure 1A, allowing clearer visualization and direct visual comparison of tracer signal distributions across the different experimental groups. This modification improves clarity and facilitates interpretation of the imaging data.

**Fig. 1** *In vivo* distribution and proposed mechanisms of ²¹¹At organ accumulation. (a) Representative whole-body micro-SPECT/CT images of BALB/c mice (n = 3 per group) acquired at 2 h after intravenous administration of free ¹³¹I (100 µCi), ¹³¹I + ascorbic acid (¹³¹I + AA), free ²¹¹At (50 µCi), and ²¹¹At + ascorbic acid (²¹¹At + AA), arranged from left to right. Images are displayed using radionuclide-specific intensity scales (Bq/mL), as indicated by the corresponding colour bars. Because ¹³¹I and ²¹¹At were acquired on different SPECT systems, signal intensities are not intended for direct quantitative comparison between radionuclides. (b) Schematic illustrating the proposed organ-specific mechanisms of off-target ²¹¹At deposition, including reactive oxygen species (ROS)-driven oxidation to cationic species (e.g., AtO⁺) in the lungs; hydrolysis and colloidal formation in the liver and spleen; and acidic hydrolysis to At⁺ in the stomach.

4. *The caption of Figure 1A should be expanded to provide full experimental details. At present, it only states that free ²¹¹At was injected and does not specify the administration of ¹³¹I, the number of animals used, time of acquisition, etc.*

**Response:**

We appreciate the reviewer’s suggestion to improve figure clarity. The caption of Figure 1 has now been substantially expanded to explicitly specify: (i) administration of both ¹³¹I and ²¹¹At, (ii) inclusion of the ascorbic acid co-administration group (²¹¹At + AA and ¹³¹I+AA), (iii) the number of animals per group (n = 3), and (iv) the imaging time point (2 h post-injection). These additions provide complete experimental context directly within the figure legend and enhance interpretability.

5. *The captions for Figure 2 would benefit from additional detail, including the number of replicates and the statistical analysis performed.*

**Response:**

We thank the reviewer for this helpful recommendation. The caption of Figure 2 has now been revised to explicitly report the number of animals per time point (n = 3) and data presentation format (mean ± SD). The caption of Figure 2 has now been revised to report the number of animals per time point (n = 3) and the data presentation format (mean ± SD). The general approach to data presentation and analysis has also been clarified in Section 2.10. These revisions improve transparency and allow readers to better assess data reliability and variability.

6. *The intensity scale bar is missing in Figure S2 and should be added.*

**Response:**

We thank the reviewer for noting this omission. Intensity scale bars have now been added to all transverse SPECT/CT images in Figure S2 to enable direct visual comparison of signal levels. In addition, the figure caption has been corrected to accurately reflect the image order and experimental conditions. These revisions improve clarity and consistency between the images and their description.

**Fig. S2** Representative transverse micro-SPECT/CT images of BALB/c mice (n = 3 per group) showing the distribution of ²¹¹At in the liver, lung, stomach, and thyroid at 2 h after intravenous injection. The upper row shows mice injected with free ²¹¹At, whereas the lower row shows mice co-injected with ²¹¹At and sodium ascorbate (²¹¹At + AA). Reduced signal intensity is observed in several transverse sections following co-administration of sodium ascorbate. Images are displayed using the same intensity scale (Bq/mL) within this figure, as indicated by the corresponding colour bar.

7. *The rationale for using fetal bovine serum (FBS) in the in vitro experiments should be clarified, given that the in vivo studies were performed in mice. The use of mouse serum may be more appropriate and should be discussed.*

**Response:**

We thank the reviewer for this important comment. In the revised manuscript, we have added an explicit rationale and limitation statement in the Discussion. Briefly, FBS was selected as a readily available, well-standardized protein-rich matrix that enables reproducible *in vitro* assessment of redox-driven speciation, protein association, and fractionation behaviour of ²¹¹At under controlled conditions. Our objective in these assays was to interrogate chemical transformation pathways (e.g., loss of At⁻, emergence of neutral/cationic and hydrophobic/macromolecular-associated fractions) rather than to reproduce species-specific pharmacokinetics.

We agree that murine serum would provide closer physiological matching to the *in vivo* model and may influence kinetics and binding equilibria. Accordingly, we now acknowledge in the Discussion that serum origin can affect quantitative distribution among fractions, and we have added a statement that future work will include validation in mouse serum to further strengthen translational relevance. Importantly, despite the use of FBS, the observed *in vitro* speciation trends are consistent with the *in vivo* imaging/biodistribution patterns (i.e., reduced anionic fraction and increased neutral/cationic and higher-molecular-weight associated species under oxidative/physiological conditions), supporting the use of FBS as a mechanistic proxy in this study.
